# Test–retest reliability of reinforcement learning parameters

**DOI:** 10.3758/s13428-023-02203-4

**Published:** 2023-09-08

**Authors:** Jessica V. Schaaf, Laura Weidinger, Lucas Molleman, Wouter van den Bos

**Affiliations:** 1 Department of Psychology, University of Amsterdam, Amsterdam, the Netherlands; 2 Cognitive Neuroscience Department, Radboud University Medical Centre, Nijmegen, the Netherlands; 3 Donders Institute for Brain, Cognition and Behaviour, Nijmegen, the Netherlands; 4 DeepMind, London, United Kingdom; 5 Center for Adaptive Rationality, Max Planck Institute for Human Development, Berlin, Germany

**Keywords:** Reinforcement learning, Computational modeling, Test–retest reliability, Computational psychiatry, Computational phenotyping

## Abstract

It has recently been suggested that parameter estimates of computational models can be used to understand individual differences at the process level. One area of research in which this approach, called computational phenotyping, has taken hold is computational psychiatry. One requirement for successful computational phenotyping is that behavior and parameters are stable over time. Surprisingly, the test–retest reliability of behavior and model parameters remains unknown for most experimental tasks and models. The present study seeks to close this gap by investigating the test–retest reliability of canonical reinforcement learning models in the context of two often-used learning paradigms: a two-armed bandit and a reversal learning task. We tested independent cohorts for the two tasks (*N* = 69 and *N* = 47) via an online testing platform with a between-test interval of five weeks. Whereas reliability was high for personality and cognitive measures (with ICCs ranging from .67 to .93), it was generally poor for the parameter estimates of the reinforcement learning models (with ICCs ranging from .02 to .52 for the bandit task and from .01 to .71 for the reversal learning task). Given that simulations indicated that our procedures could detect high test–retest reliability, this suggests that a significant proportion of the variability must be ascribed to the participants themselves. In support of that hypothesis, we show that mood (stress and happiness) can partly explain within-participant variability. Taken together, these results are critical for current practices in computational phenotyping and suggest that individual variability should be taken into account in the future development of the field.

**Supplementary Information:**

The online version contains supplementary material available at 10.3758/s13428-023-02203-4.

## Introduction

The use of computational models to mathematically formalize and describe the cognitive processes underlying learning has become increasingly popular (Palminteri et al., 2017), especially models derived from reinforcement learning theory. This theory offers a set of formal models for learning from feedback, where feedback (a reinforcer) is used to update beliefs about the outcome of future decisions (Q values; Daw, 2011; Sutton & Barto, 2018). Central to the theory is the reward prediction error: the difference between current beliefs and the experienced outcome associated with an action. In the past few decades, a clear link between the dopamine system and this reward prediction error has been established in studies of both humans and animals (e.g., Niv, 2009), providing a bridge between brain and behavior. The extent to which the prediction error is used to update beliefs is dependent on (1) the magnitude (and sign) of the prediction error, and (2) the learning rate (see Section "The models"). Computational models can be used to estimate the best-fitting learning rate for each individual based on their decisions on a learning task. In this context, model parameters, like the learning rate, can be compared between groups or individuals to characterize underlying learning processes.

One of the fields in which reinforcement learning theory is increasingly applied is computational psychiatry (Adams et al., 2016; Friston et al., 2014; Huys et al., 2011, 2016; Maia & Frank, 2011; Montague et al., 2012; Paulus et al., 2016; Petzschner et al., 2017; Stephan et al., 2017; Wang & Krystal, 2014). The reason for this is that many psychiatric disorders are associated with deficits in learning, suggesting aberrant functioning of the dopamine system (Montague et al., 2012; Schultz et al., 1997). On a group level, there is evidence for aberrant processing of prediction errors in various psychiatric disorders, including psychosis (Corlett et al., 2007; Murray et al., 2008), obsessive–compulsive disorder (Hauser et al., 2017), ADHD (Hauser et al., 2014), depression (Gradin et al., 2011), gambling addiction (Linnet, 2014), and substance abuse (Tanabe et al., 2013). However, the results on learning rates are mixed, with several papers reporting differences in learning rates in relation to anhedonia, schizophrenia, impulse control, and autism (Chase et al., 2010; Insel et al., 2014; Lin et al., 2012; Piray et al., 2014), but a similar number of studies not observing differences between clinical groups (e.g., Gradin et al., 2011; Linnet, 2014; Murray et al., 2008). These initial findings are promising and show the potential of using computational reinforcement learning models for understanding psychiatric disorders and other individual differences.

Building on this potential, individual model parameters have been used as indicators of psychiatric disorders, so-called cognitive phenotyping (Patzelt et al., 2018). The promise of this approach is to provide a more detailed understanding of cognitive phenotypes on an individual level, going beyond mere symptomology, and to provide a bridge between brain and behavior. Together, this is thought to help explain the causes of psychiatric disorders, and to improve diagnosis and treatment. However, in order to use individual model parameters for cognitive phenotyping, there are still several key challenges to be met (Eckstein et al., 2021; Patzelt et al., 2018; Stephan et al., 2017).

One of the main unmet challenges, and the focus of this paper, is that very little is known about the stability of the learning parameters over time. As recently pointed out by Patzelt et al. (2018), the “test–retest reliability [of the parameter estimates] will be especially important for establishing the utility of phenotypes in predicting clinical outcomes and treatment development as we move from translational neuroscience to clinical application” (see also Eckstein et al., 2021; Palminteri & Chevallier, 2018; Paulus et al., 2016; Stephan et al., 2017). Stability of parameters is central to the computational phenotyping endeavor. That is, reinforcement learning tasks should replicate the same ordering between participants (reliability) and should ideally provide strictly identical results (agreement) when participants are measured twice (Berchtold, 2016). However, only a few very recent studies have addressed the test–retest reliability of the reinforcement learning parameters used for computational phenotyping (Brown et al., 2020; Loosen et al., 2022; Waltmann et al., 2022). In general, these studies found poor to moderate test–retest reliability for model parameters in a reversal learning task (Waltmann et al., 2022; reliabilities for learning rates ranging from .16 to .59 across different parameter estimation methods) and a sequential reinforcement learning task (Brown et al., 2020; reliabilities for learning rates ranging from −.20 to .95 across different data cleaning and parameter estimation methods, but between .40 and .45 on average). However, good test–retest reliability was found in a predictive-inference task in which learning rates were derived directly from observed predictions (Loosen et al., 2022; reliabilities ranging from .74 to .82).

**Fig. 1:**
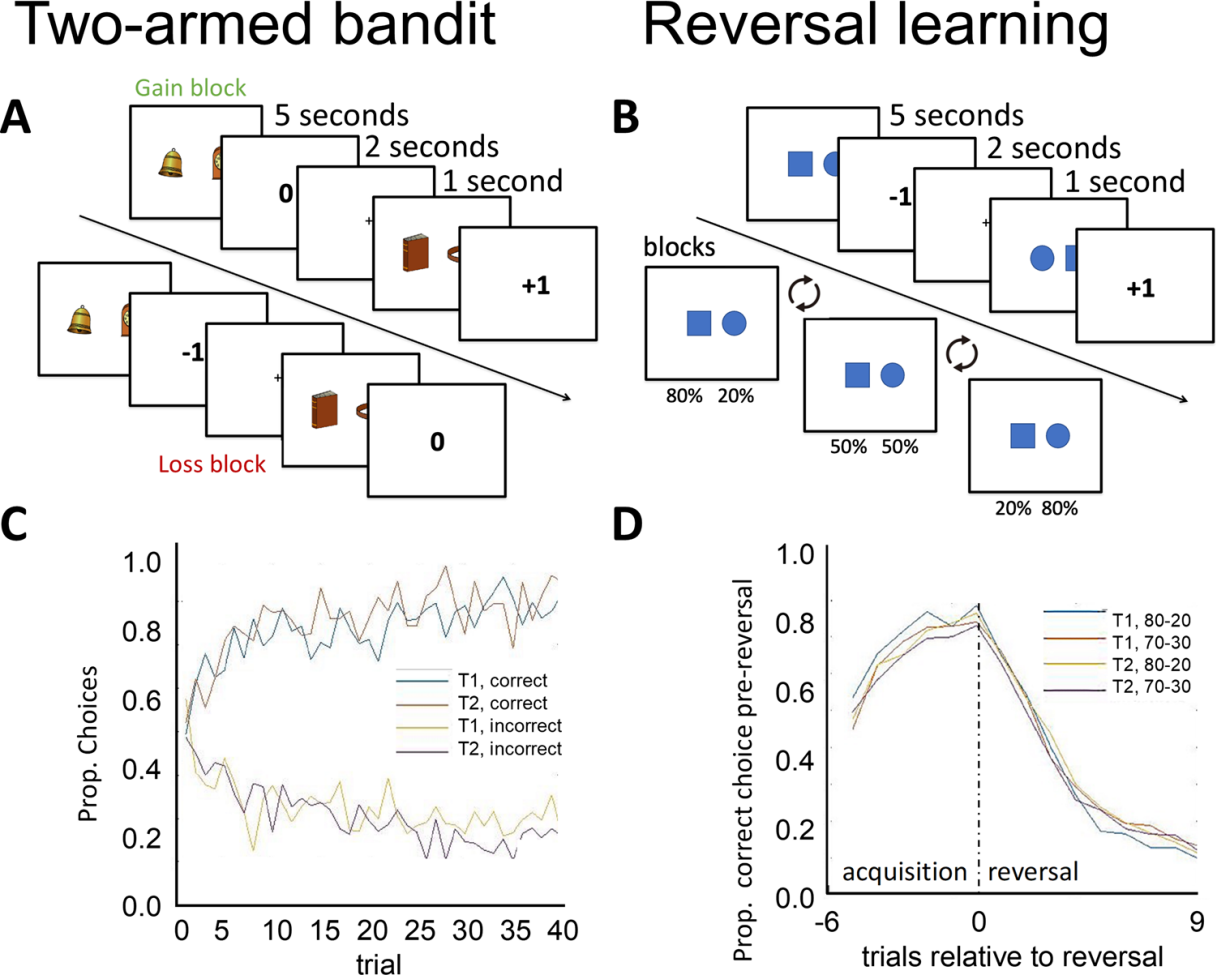
Example trials of the two reinforcement learning tasks. *Note*. **A**: two trial sequences of the two-armed bandit task. The top sequence (the gain block) shows how a simulated participant first chooses between the bell and the clock stimulus and gains 0 points (indicating an incorrect choice) and then chooses between the book and the ring and gains 1 point (indicating a correct choice). The bottom sequence (the loss block) shows how a participant first loses 1 point (indicating an incorrect choice) and then loses 0 points (indicating a correct choice). **B**: trial and block sequences of the reversal learning task. The top sequence (the trial sequence) shows how a simulated participant chooses between the square and the circle and loses a point and then, choosing between the same stimuli, gains a point. The bottom sequence (the block sequence) shows how the correct stimulus (the stimulus with the highest reward probability) changes across blocks. Note stimuli are schematic for illustration purposes, see supplement for more details and instructions. **C**: Trial-by-trial learning curves in the bandit task, that is, per trial the proportion of participants that chose the “correct” stimulus (associated with a probability of .8 of gaining money) in the gain condition (upper lines), and the “incorrect” stimulus (associated with a probability of .8 of losing money) in the loss condition (cf. Pessiglione et al., 2006). **D**: Trial-by-trial learning curves in the reversal learning task relative to the reversal, that is, the proportion of participants that chose the “correct” stimulus (associated with a probability of .7 or .8 of gaining money) before the reversal (acquisition phase) and the proportion of participants that keep choosing this – now “incorrect” – stimulus after the reversal (reversal phase)

Here we will investigate the test–retest reliability, over a period of five weeks, of learning parameters from two often-used learning tasks in computational psychiatry and aging research (see Fig. 1): a two-armed bandit task (Frank et al., 2004; Pessiglione et al., 2006) and a reversal learning task (Cools et al., 2002; Schlagenhauf et al., 2014) comparing more traditional model fitting procedures with more recently developed hierarchical and joint Bayesian modeling. Although moderate reliability of behavioral measures was previously found in a two-armed bandit task (Pratt et al., 2021; reliability of .63), the reliability of reinforcement learning parameters in this task is currently unknown. In addition, we try to replicate the poor to moderate parameter reliability as reported by Waltmann et al. (2022).

Staying close to what is reported in the majority of the computational psychiatry literature, we will report the results of parameter estimates of a set simple Rescorla–Wagner-type reinforcement learning models. We are not so much interested in which model provides the best fit to the data, but more in how model complexity influences test–retest reliability. In the same vein, we will compare different model fitting techniques to see how these affect reliability. Specifically, we will compare the most common approach (i.e., maximum likelihood estimation, MLE) to regularized estimation methods (i.e., maximum a priori estimation, MAP, and hierarchical Bayesian modeling, hBayes), as these methods are shown to improve parameter identifiability and stability (Daw, 2011; Gershman, 2016; Spektor & Kellen, 2018; Wagenmakers et al., 2016). In addition, we benchmark our test–retest estimates against brief personality and cognitive ability measures, and simulations. Finally, to characterize potential variation in parameters across time points, we focus on a source of variance known to vary over time and to relate to learning rates (Aylward et al., 2019; Bakic et al., 2014; Kube et al., 2023; Paul et al., 2020; Paul & Pourtois, 2017), that is, we asked participants to report on current mood. Both behavioral tasks were incentivized and administered online in independent samples.

## Method

### Participants

Participants located in the United States were recruited via the Amazon Mechanical Turk online testing platform and performed the tasks with a between-test interval of five weeks. All participants were informed about the two-session nature of the study and that payment could only be acquired after completing both sessions. Participants who performed the task on time point 1 (T1) were invited to retake the task five weeks later. For each task, 150 participants were invited, and participants could only take part in one of the tasks. This number was not preregistered, but based on effect sizes and drop-out rates in a previous test–retest reliability study we ran (Molleman et al., 2019). Based on this information, we argued that inviting 150 participants would leave us with a sample size feasible for psychiatric studies while being able to detect potentially small effects. The two-armed bandit task was completed by 142 participants during T1 and 93 during T2. The reversal learning task was completed by 154 (four more due to technical error) during T1 and 64 during T2. We excluded participants who failed to provide a valid MTurk ID, who timed out on more than 20% of trials, and who commented after completing the task indicating that they misunderstood the task. Additionally, we excluded participants when overall accuracy dropped below 55% (cf., Waltmann et al., 2022), as this would result in unreliable parameter estimates. Finally, we only included participants who met inclusion criteria during both T1 and T2, participants we coin “returners.” This resulted in 69 participants (25 female, *M*_age_ = 35, *SD*_age_ = 11) for the bandit task and 47 for the reversal learning task (23 female, *M*_age_ = 39, *SD*_age_ = 12). For a complete description of the excluded participants, we refer to Table S8.

### Reinforcement learning tasks

#### Two-armed bandit

Following Pessiglione et al. (2006), in the two-armed bandit task, we presented pairs of pictures (everyday objects) that were associated with probabilistic monetary gains or losses. Participants repeatedly chose between the two pictures to maximize payoffs (Fig. 1A). In total, the experiment consisted of four blocks, two “gain” blocks and two “loss” blocks. Participants randomly started with either a gain or a loss block and performed the remaining blocks in alternating order (ABAB). In the gain block, the two pairs of pictures were associated with gain ($1 or nothing), in the loss blocks with loss (−$1 or nothing). Feedback was probabilistic such that the two pairs in each block had 80% (versus 20%) and 70% (versus 30%) congruent feedback. Each pair was presented 20 times, totaling 160 trials per participant.

#### Reversal learning

Following Cools et al. (2002) and Schlagenhauf et al. (2014), in the reversal learning task, we presented a single pair of geometric shapes (circle and square) of which participants chose one to maximize their payoff (Fig. 1B). On each trial, participants could either gain or lose a dollar (+$1 or −$1). The reversal learning task consisted of three different states associated with different reward probabilities (80% vs. 20%, 20% vs. 80% and 50% vs. 50%). A switch between these states (reversal) would occur when the participant chose the most rewarding option 7 out of 10 times in the last 10 trials, or when 16 trials passed in one state. For the 50/50 reward state, the “most rewarding” stimulus was set to the least rewarding stimulus in the last reward state (i.e., the stimulus with a reward probability of 20% in the preceding state) in order to determine when a reversal should occur. The task ended after 250 trials regardless of the number of reversals. Participants were informed about the reversals (“Throughout the task it may change multiple times which symbol is more likely to win and which is more likely to lose”), but not about when these reversals would occur. They were also informed about the probabilistic nature of the feedback (“It can happen that even though you choose the symbol that was more likely to win, you lose.”).

#### Incentives

Both learning tasks were incentivized. All participants started with a bonus of $1 and were told that they could earn a bigger bonus or lose the bonus depending on their choices. We instructed participants that each $1 (gained or lost) corresponded to $0.05 (gained or lost) in bonus payment, and that failing to respond in time would result in losing $0.05. They were also informed that they could earn up to $12.50.

#### Cognitive ability, personality, and mood measures

After the learning tasks, we administered several questionnaires to measure individual differences in cognitive ability, personality, and mood. First, we included a short measure of general cognitive ability: the Raven progressive matrices of visuospatial reasoning (Raven, 1941). For each time point we included ten exercises of different difficulty levels. Next, participants performed an n-back task (Kirchner, 1958), measuring working memory capacity, which is not analyzed for the purpose of this paper. Then*,* to measure personality, we administered the Mini-IPIP, a 20-item short form of the 50-item International Personality Item Pool-Five-Factor Model measure (Donnellan et al., 2006). Finally, to assess current mood, we asked participants to answer, on a Likert scale from 1 (completely disagree) to 5 (completely agree), whether they currently felt tired, happy, hungry, stressed, awake, worried, bored, and relaxed.

### Behavioral analysis

We analyzed three behavioral measures commonly reported in the computational psychiatry field: accuracy, and the probabilities to win-stay and lose-shift. Timed-out trials and trials with 50/50 reward probability (only in reversal learning task) were excluded from the accuracy calculations, given that a normatively right or wrong answer was lacking. Accuracy is defined as the number of times the stimulus with a higher reward probability was chosen, divided by all trials. Win-stay is the proportion of trials where participants chose the same stimulus as on the previous trial following positive feedback and lose-shift is the proportion where they chose the opposite stimulus following negative feedback.

To assess the test–retest *reliability* of behavioral measures we used Pearson’s correlation, and to assess the test–retest *agreement* we used the intra-class correlation coefficient (ICC (3,1)). Following Waltmann et al. (2022), we also included the ICC derived from mixed-effects models that included both the T1 and T2 datasets, ($alpha \sim 1+\left(1|ID\right)+(1|session$)). We interpreted all reported ICC(3,1) coefficients following Koo and Li (2016), with *r* < .5 indicating “poor,” .5 ≤ *r* < .75 “moderate,” .75 ≤ *r < *.9 “good,” and *r* ≥ .9 “excellent” reliability. When reporting the ICCs for the cognitive ability, personality and mood measures, behavioral task measures and computational parameters fit to the empirical data, we also report the between-session variance and the error variance between brackets (ICC [between-session variance/residual variance]), to take into account potential session differences (e.g., practice effects, see Hedge et al., 2018).

### Computational modeling

#### The models

As reinforcement learning theory is the most prominent theory used for computational phenotyping, we fitted reinforcement learning algorithms to participants’ choices to infer underlying parameter values (Sutton & Barto, 2018). Given that the aim of this paper was to provide additional insight into how model complexity may impact the reliability and agreement of parameter estimates, we focus on a subset of reinforcement learning models commonly applied in the computational phenotyping field. Specifically, we applied different variants of the Rescorla–Wagner model (Rescorla & Wagner, 1972). In this model, choices result from a trial-by-trial (*t*) calculation of beliefs about the outcome (*Q*) of a choice (*c*: left or right, see Fig. 1), weighed by prediction errors (*δ*) and the learning rate (0 ≤ *α* ≤ 1).

$${Q}_{c,t+1}={Q}_{c,t}+\alpha {\cdot \delta }_{t}$$

The prediction error constitutes the trial-by-trial mismatch between the current belief about the outcome of the choice and the observed reward (*r*).

$${\delta }_{t}={r}_{t}-{Q}_{c,t}$$

We initialized all models with ${Q}_{c,t=0}$ values of zero. Finally, we used a standard softmax function to generate trial-by-trial probabilities of the observed choices.

$${p}_{c=left,t}=\frac{1}{{e}^{-\tau ({Q}_{c=left,t}-{Q}_{c=right,t})}}$$

where *τ* is the free parameter capturing decision noise (0 ≤ *τ* ≤ 20). We extended this basic algorithm in two ways.

##### Dual learning rates

One addition to the basic algorithm is implementing separate learning rates for gains (*α*_*gain*_) and losses (*α*_*loss*_; Kahnt et al., 2009; van den Bos et al., 2012). These models are referred to as dual-learning-rate models and model an asymmetry between how people learn from gains and losses. It has been hypothesized that, depending on the distribution of reward probabilities, asymmetric learning rates for gains and losses can be adaptive (Cazé & Van Der Meer, 2013). Accordingly, dual-learning-rate models often fit data from simple bandit tasks better than the basic algorithm. Also, several studies have identified individual differences in learning from gains and losses (e.g., Eppinger & Kray, 2011; Frank et al., 2005), potentially due to differences in sensitivity to gains (Carver & White, 1994).

##### Double updating

Another addition is implementing that learners update values of both the chosen and unchosen option (Reiter et al., 2016), exploiting the task characteristic that reward probabilities of the options in a pair are anticorrelated (i.e., when one is high the other is low). These models are referred to as the double-update (DU) models, and code the reward of the unchosen option as the opposite of the chosen option. For the bandit task reward, recoding is done block-wise:

$$r_{unchosen}=\left\{\begin{array}{cc}1,&r_{chosen}=0\;and\;gain\;block\\0,&r_{chosen}=1\;or\;r_{chosen}=-1\\-1,&r_{chosen}=0\;and\;loss\;block\end{array}\right.\\$$

whereas for the reversal learning task, the rewards are simply multiplied by −1:

$${r}_{unchosen}=-1{\cdot r}_{chosen}$$

To update the value of the unchosen option (*Q*_*unchosen*_) a separate prediction error is calculated:

$${\delta }_{unchosen,t}={r}_{unchosen,t}-{Q}_{unchosen,t}$$

We implemented two variants of double updating: a full DU model and a partial DU model. In the full DU model, the value of both the chosen and unchosen option are updated to an equal degree, that is, with the same learning rate. In the partial DU model, the value of the unchosen option is updated to a lesser degree than the value of the chosen option, which is implemented by a linear transformation of the learning rate:

$${Q}_{unchosen,t+1}={Q}_{unchosen,t}+\kappa \cdot \alpha {\cdot \delta }_{unchosen,t}$$

where $\kappa$ (0 ≤ $\kappa$ ≤ 1) is a free parameter that down-weights the update of the value of the unchosen option. A $\kappa$ value of 0 indicates no double updating, whereas a $\kappa$ value of 1 indicates equal updating of the chosen and unchosen option. In the following, we refer to this set of models as the kDU models. The complete model space consisted of all combinations of learning rates and update rules, totaling six different models.

#### Model fitting procedure

A secondary aim of this paper was to assess how model fitting procedures affect the reliability and agreement of reinforcement learning parameters. We therefore compared standard procedures in the computational phenotyping field (maximum likelihood estimation) to regularized procedures (maximum a priori and hierarchical Bayesian estimation) shown to improve parameter reliability in other contexts.

##### Maximum likelihood estimation (MLE)

Following standard procedures, we fitted our models using maximum likelihood estimation, that is, maximizing the logarithm of the probability of the data ($$D$$; i.e., participants’ choices) given a model ($$M)$$ and a set of parameter values ($${\theta }_{M})$$, thus $$P\left(D|M,{\theta }_{M}\right).$$ All model fitting was done using the general-purpose optimization toolbox (*optim*) in the R programming language (R Core Team, 2018), with the “L-BFGS-B” quasi-Newton method which allows each variable to be given a lower and/or upper bound (as reported in Section "The models"). Timed-out trials were excluded from computational modeling as participants did not receive feedback at these trials, making belief updating impossible. Each model was fitted 20 times per participant with random initial parameter values to prevent getting stuck in local minima. Analysis code is shared on the Open Science Framework (https://osf.io/pe23t/). Model selection was performed based on the Bayesian information criterion (BIC; Schwarz, 1978).

##### Maximum a posteriori estimation (MAP)

Maximum a posteriori (MAP) estimation uses priors over the distributions of the parameter values $, P\left({\theta }_{M}|M\right),$ to regularize the parameter estimates during the fitting. It has been suggested that, in the right circumstances (Spektor & Kellen, 2018), using these priors can result in more reliable and stable parameter estimates than standard MLE methods (Daw, 2011). These priors can be informed by either previous studies with similar tasks, or by an initial MLE fit on the data (note that MLE is equal to MAP with uninformed priors). In each case the distribution of parameter values is estimated from a population. In our models the priors are implemented as beta distributions for parameters that are bounded between zero and 1 ($\alpha ,\;{\alpha }_{gain},\;{\alpha }_{loss}, \;and \;\kappa )$. For the model fitting at T1 we have used the a posteriori estimated distributions and refit the model. For T2, we have compared using the a posteriori distributions of both T1 and those of T2. Note that using the priors based on the a posteriori parameter distributions is similar (but not the same) as the hierarchical Bayesian method in the sense that it uses information about the group to regularize the parameter estimates of the individuals in that same group.

##### Hierarchical Bayesian estimation (hBayes)

Finally, we used hierarchical Bayesian estimation to obtain more reliable parameter estimates by using group distributions to bound participants’ parameter estimates (Efron & Morris, 1977) and by incorporating uncertainty in the estimation process (Lee & Wagenmakers, 2013; Wagenmakers et al., 2016). For the model fitting at T1 we implemented uninformed priors on all group parameters (i.e., mean and precision of $\alpha ,\;{\alpha }_{gain},\;{\alpha }_{loss}, \;and\; \kappa$): group-level mean values were bounded between 0 and 1, group-level precision values between 2 and 600 (Steingroever et al., 2014). Participants’ parameters were implemented as beta distributions obtained from these group distributions. At T2, we fitted the models using the same uninformed priors as at T1, but also using informed priors based on the obtained distributions at T1. All models were fit in JAGS (Plummer, 2003) using the R2jags package (Su & Yajima, 2015). We determined which model fitted the data best using the deviance information criterion (DIC; Spiegelhalter et al., 2002), a model fit index designed for complex hierarchical models.

## Results

### Behavioral results

#### Accuracy

To investigate whether participants performed the tasks adequately, we first inspected accuracy (defined as the percentage of choices for the stimulus with the highest reward probability). As illustrated in Fig. 1C, in the bandit task, participants achieved mean accuracy of 70.4% (SD = 10.4%) for T1 and 72.6% (SD = 8.6%) for T2. As illustrated in Fig. 1D, in the reversal learning task, participants achieved mean accuracy of 65.7% (SD = 9.4%) for T1 and 57.0% (SD = 8.1%) for T2. In all cases, accuracy was similar for T1 and T2 (*p*s > .30).

#### Reliability and agreement of behavioral task measures

**Fig. 2:**
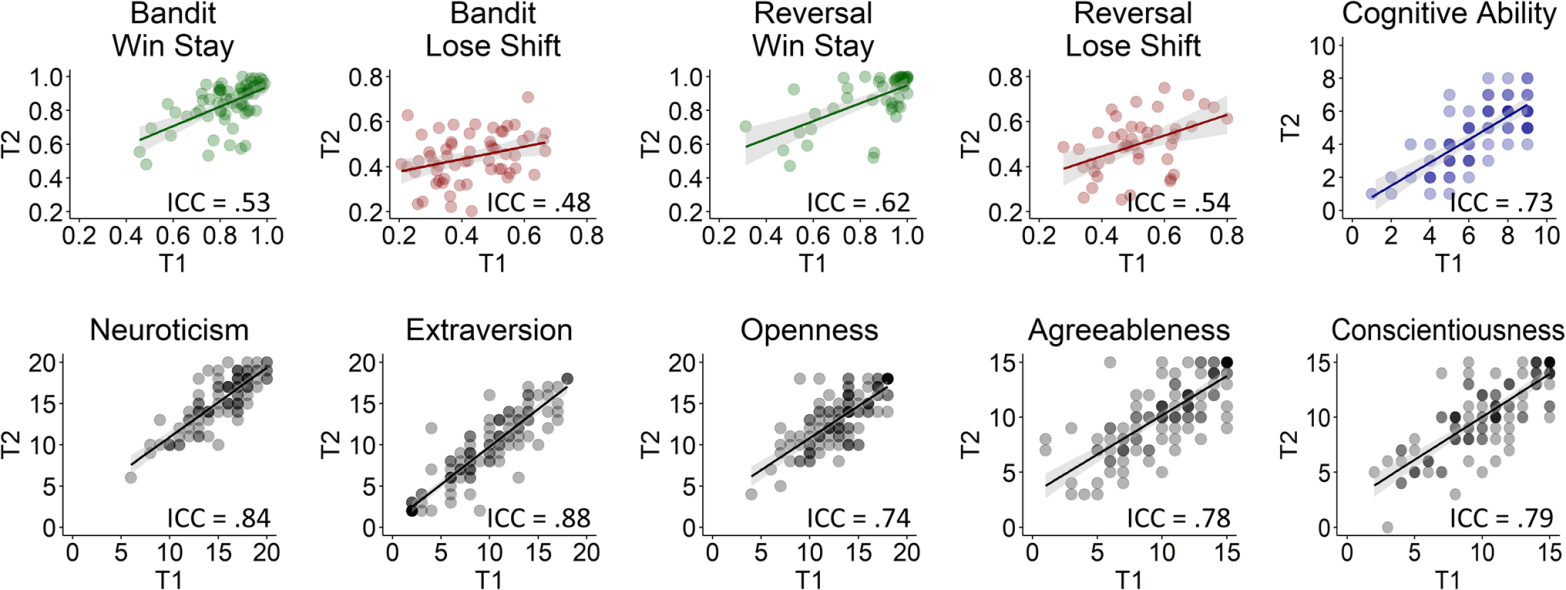
Reliability and agreement between T1 and T2 for shifting strategies, cognitive ability, and personality traits. Top row, first four panels: shifting strategies showed moderate to good reliability for both tasks. Top row, right panel: cognitive ability showed good reliability. Bottom row: personality traits showed good to excellent reliability.

We then assessed the reliability and agreement of the three commonly reported behavioral measures (accuracy, win-stay, and lose-shift). In the bandit task, reliability of accuracy was small but significant (*r = *.28), and the ICC was poor (ICC = .23 [session = .00/error = .77])*.* In contrast, the shifting strategies showed medium Pearson’s correlation coefficients and moderate ICC scores; win-stay: *r = *.53 and ICC = .53 [.00/.47]; lose-shift: *r* = .48 and ICC = .48 [.01/.51]. We observed a similar, albeit slightly more promising, pattern in the reversal learning task (see Fig. 2). Here, Pearson’s correlation coefficient for accuracy was medium (*r* = .50), and the ICC was moderate (ICC = .50 [.00/.50]). And again, shifting strategies showed better reliability, with medium Pearson’s correlation coefficients and moderate to good ICC scores; win-stay: *r* = .63 and ICC = .62 [.00/.38]; lose-shift: *r* = .55 and ICC = .54 [.00/.46]. In sum, shifting strategies showed moderate to good reliability, and the reliability for the reversal learning task was higher than for the bandit task.

### Mood, personality, and cognitive ability results

#### Mood

Most of the mood measures showed moderate reliability and agreement over the five-week period. The Pearson correlations ranged from .37 to .77 and the ICCs ranged from .37 (poor) to .77 (good; for more detailed results see Table S1). Here, the least reliable measure was hunger, whereas the most reliable was feeling relaxed. Given that some mood states appeared to be (anti-)correlated, we performed exploratory factor analyses with oblique rotation to reduce dimensionality for subsequent analyses, resulting in three factors (stress, wakefulness, and happiness; see Table S2).

#### Personality and cognitive ability

As expected, the IPIP big five personality scales showed high test–retest reliability and good to excellent agreement, with Pearson correlations ranging from .68 to .93 and ICCs between .67 and .93 (see Fig. 2 and Table S1 for more detailed results). Like personality, cognitive ability (operationalized as accuracy on the Raven test) showed the expected high reliability (*r* = .60) and good agreement (ICC = .60). In sum, the two trait measures performed as expected in terms of reliability and agreement in our sample and provide a benchmark for evaluating the reliability of the task-based measures.

### Computational modeling results

#### Two-armed bandit

In concordance with previous findings, the model comparison results showed a consistent pattern of the dual-learning-rate models outperforming the single-learning-rate models. However, it was unclear whether including weighted double updating contributed to a better fit (see Table 1). Given that the aim of this paper was to provide additional insight into how model complexity may impact the reliability and agreement of parameter estimates, and not to identify the best model, we report on both models (dual RL and dual RL kDU). Interestingly, using the empirical prior based on T1 to regularize T2 model fitting, using MAP or hBayes, did not improve model fit compared to without such empirical priors (see Table S3 for MAP priors used).

**Table 1:** Model fit indices per time point (T1 and T2) for all six models across the three fitting procedures (MLE, MAP, hBayes) for the bandit task

	BIC (MLE)		BIC (MAP)			DIC (hBayes)		
	T1	T2	T1(T1)	T2(T1)	T2(T2)	T1	T2(T1)	T2
Simple RL	10,050	9988	11,060	11,115	11,114	10,168	10,212	10,233
Simple RL DU	11,509	11,094	12,080	12,499	12,490	12,757	11,866	11,878
Simple RL kDU	9882	9693	11,289	11,427	11,472	9920	9967	9993
Dual RL	9340	9397	**11,016**	**10,926**	**10,927**	9700	9608	9616
Dual RL DU	10,795	10,446	11,935	12,320	12,259	9621	9867	9886
Dual RL kDU	**9135**	**9098**	11,225	11,221	11,249	**9181**	**9197**	**9233**

For MAP and hBayes, the data used for the prior are indicated within brackets. For all indices holds: the lower, the better the model fits the data. Best-fitting models (i.e., with the lowest fit indices) are indicated in boldface

When comparing the parameter estimates for the best-fitting models (dual RL and dual RL kDU) generated by MAP and hBayes, we found that these were highly correlated (all *r*s > .9 see Table S4) for the dual RL model, and less so for the dual RL kDU model (but still strong; most *r*s > .7, with the exception of parameter $\kappa ;$ see Table S4), suggesting that the two regularization methods converge on similar solutions.

##### Parameter reliability

The first striking result is that the parameter reliability and agreement for the MLE fitted models is extremely poor for all parameters, except for inverse temperature ($\tau$) in the dual RL kDU model (see Table 2). Although, at least for the dual RL model, reliability and agreement are better for MAP and hBayes, for all parameters except the $\tau$, reliability and agreement still qualify as poor. For the dual RL kDU model we found a similar pattern of results.

**Table 2:** Reliability and agreement of the parameter estimates from the dual RL and dual RL kDU models in the bandit task for the three fitting procedures

	$\tau$		${\alpha }_{gain}$		${\alpha }_{loss}$		*κ*	
	*r*	ICC	*r*	ICC	*r*	ICC	*r*	ICC
MLE								
Dual RL	.07	.08 [.00/.92]	.11	.12 [.00/.88]	.16	.16 [.00/.84]	-	-
Dual RL kDU	.33	.33 [.01/.66]	.15	.11 [.00/.89]	.12	.12 [.00/.88]	.12	.12 [.00/.88]
MAP								
Dual RL	.38	.38 [.00/.62]	.24	.24 [.00/.76]	.20	.21 [.00/.79]	-	-
Dual RL kDU	.52	.52 [.00/.48]	.18	.16 [.01/.83]	.02	.02 [.00/.98]	.22	.23 [.00/.77]
hBayes								
Dual RL	.41	.42 [.00/.58]	.22	.22 [.03/.75]	.21	.22 [.00/.78]	-	-
Dual RL kDU	.46	.46 [.00/.54]	.18	.16 [.03/.81]	.27	.27 [.00/.73]	.25	.18 [.10/.72]

Inverse temperature (*τ*), learning rate (*α*), and update parameter (*κ*). Pearson correlations (*r*) and ICC(3,1). Values between square brackets indicate proportion of between-session variance and error variance

##### Internal validity

Finally, we gauged the internal validity of the parameter estimates of the models by estimating how *α*_*gain*_ and the win-stay probability and how *α*_*loss*_ and the lose-shift probability relate. Given that we have two dependent observations that contribute to the correlation between learning rates and shifting strategies, we tested this over the two sessions in a mixed-model beta-regression analysis with measures of sessions 1 and 2 nested in participants as random effects using the glmmADMB package (Skaug et al., 2018).

For the dual RL model, we found the expected positive relationship between sessions across all model fitting procedures. However, for the dual RL kDU model, internal validity was poor under MLE and MAP regularization but showed more promising results under hBayes (see Table 3). As such, these results suggest that the hBayes method outperforms the other methods when estimating the parameters of more complex models.

**Table 3:** Results of beta-regression on learning rates and shifting strategies in the bandit task

	${\alpha }_{gain}$*, win-stay*	${\alpha }_{loss}$*, lose-shift*
	$\beta$	$\beta$
MLE		
Dual RL	0.10 (0.02)***	0.51 (0.31)
Dual RL kDU	0.04 (0.02)	0.09 (0.05)
MAP		
Dual RL	0.18 (0.04)***	0.29 (0.10)***
Dual RL kDU	0.06 (0.04)	0.06 (0.11)
hBayes		
Dual RL	0.20 (0.04)***	0.15 (0.06)***
Dual RL kDU	0.32 (0.05)***	0.27 (0.05)***

Standard errors are indicated between brackets; *** *p* < .001

##### Parameter identifiability

A prerequisite of test–retest reliability is parameter identifiability. To assess such parameter identifiability in our model set, and to get an idea of the range of measures we could expect if people’s parameters (${\alpha }_{gain}$, ${\alpha }_{loss}$, *κ*) would be perfectly stable over time (and what could be ascribed to the stochasticity inherent to the model behavior ($\tau$) and other sources of noise), we performed parameter identifiability analyses on a representative set of 100 simulated participants. The parameter values of the simulated participants were based on distributions as found in our current data set (T1)^1^: each simulated participant consists of a set of parameters drawn from the distributions that we used for the T1 MAP analyses. Next, we simulated behavior in context of the experiment that uses the exact same settings and number of trials as our online experiments. We generated two data sets for each simulated participant, representing T1 and T2 measures (ground truth: ${M}_{\tau }=5.25$, ${SD}_{\tau }=2.18$; ${M}_{{\alpha }_{gain}}=.70$, ${SD}_{{\alpha }_{gain}}=.23$; ${M}_{{\alpha }_{loss}}=.25$, ${SD}_{{\alpha }_{loss}}=.16$). For these analyses we again focused on the two best-fitting models (dual RL and dual RL kDU).

Across fitting procedures, we found that ${\alpha }_{gain}$ showed only moderate identifiability, whereas we found high identifiability for ${\alpha }_{loss}$ and $\tau$ for the regularized fitting procedures (see Table 4). These results are very similar to those recently presented for the more complex two-step learning task (including more complex models; Shahar et al., 2019). Overall, the levels of identifiability suggest that our procedures can detect satisfactory test–retest reliability if participants are stable over time. Of course, our simulated participants were stable, and as expected, for MAP and hBayes we find that simulations of two time points led to a good level of reliability for ${\alpha }_{loss}$ and $\tau$, but also the expected moderate reliability for ${\alpha }_{gain}$ (see Table 5). Note that the hBayes model did show poor results for the *κ* parameter for both identifiability and test–retest reliability.

**Table 4:** Simulated T1 identifiability of the parameter estimates from the dual RL and dual RL kDU models in the bandit task for the three fitting procedures

	$\tau$		${\alpha }_{gain}$		${\alpha }_{loss}$		*κ*	
	*r*	ICC	*r*	ICC	*r*	ICC	*r*	ICC
MLE								
Dual RL	.61	.55	.48	.47	.79	.77	-	-
Dual RL kDU	.69	.64	.60	.58	.79	.76	.71	.62
MAP								
Dual RL	.83	.83	.61	.61	.87	.84	-	-
Dual RL kDU	.80	.78	.67	.67	.81	.82	.66	.60
hBayes								
Dual RL	.84	.82	.63	.56	.87	.86	-	-
Dual RL kDU	.81	.79	.69	.61	.89	.89	−.08	~.01

Pearson correlations (*r*) and ICC(3,1)

**Table 5:** Simulated test–retest reliability of the parameter estimates from the dual RL and dual RL kDU models in the bandit task for the three fitting procedures

	$\tau$		${\alpha }_{gain}$		${\alpha }_{loss}$		*κ*	
	*r*	ICC	*r*	ICC	*r*	ICC	*r*	ICC
MLE								
Dual RL	.54	.55	.44	.47	.74	.74	-	-
Dual RL kDU	.56	.56	.39	.39	.49	.49	.39	.39
MAP								
Dual RL	.76	.76	.56	.56	.85	.85	-	-
Dual RL kDU	.76	.76	.55	.55	.65	.65	.55	.55
hBayes								
Dual RL	.81	.81	.63	.62	.86	.86	-	-
Dual RL kDU	.76	.76	.60	.58	.83	.83	.15	.12

Pearson correlations (*r*) and ICC(3,1)

##### Interim summary

Consistent with the literature, our simulations indicated that regularizing methods (MAP and hBayes) improve parameter identifiability. Furthermore, for the assumed parameter space, and the given experimental designs (number of trials, pairs, probabilities, etc.), this led to a level of identifiability for ${\alpha }_{loss}$ and $\tau$ that is sufficiently high to detect good reliability, if participants would apply stable learning strategies. However, the true test–retest reliability score for the learning rates of our participants remains poor at best. Potential reasons will be further explored below (Section "Exploratory analyses: Explaining variability with mood") and in the discussion.

#### Reversal learning

Consistent with the two-armed bandit results, model comparison results showed a consistent pattern of the dual-learning-rate models outperforming the single-learning-rate models. Again, it is unclear whether weighted double updating enhanced model fit (see Table 6). Note that, again, the parameter estimates generated by MAP and hBayes were highly correlated for the dual RL model, and less so for the dual RL kDU model (all *r*s > .8 and *r*s > .5 respectively; see Table S5).

**Table 6:** Model fit indices per time point (T1 and T2) for all six models across the three fitting procedures (MLE, MAP, hBayes) for the reversal learning task

	BIC (MLE)		BIC (MAP)			DIC (hBayes)		
	T1	T2	T1(T1)	T2(T1)	T2(T2)	T1	T2(T1)	T2
Simple RL	10,232	10,600	10,957	11,301	11,272	10,361	10,728	10,740
Simple RL DU	11,591	12,057	11,197	11,865	11,875	10,251	10,370	10,389
Simple RL kDU	10,162	10,630	10,911	11,433	11,440	10,602	11,332	11,371
Dual RL	10,054	**10,168**	**10,941**	**11,235**	**11,213**	10,118	10,067	10,075
Dual RL DU	10,458	11,173	11,497	12,247	12,326	10,074	10,577	10,596
Dual RL kDU	**9763**	10,177	11,036	11,319	11,431	**10,029**	**10,021**	**10,001**

For MAP and hBayes, the data used for the prior are indicated within brackets. For all indices holds: the lower, the better the model fits the data. Best-fitting models (i.e., with the lowest fit indices) are indicated in boldface

##### Parameter reliability

Similar to the bandit results, for reversal learning we found worse parameter reliability and agreement for the MLE fitted models compared to MAP and hBayes, with the exception of ${\alpha }_{loss}$ for the dual RL model (see Table 7). In addition, the ${\alpha }_{gain}$ parameter showed consistently poor reliability and agreement across all models. As compared to the bandit results, reliability and agreement for the MAP and hBayes methods were better for the reversal learning task, with moderate ICCs for ${\alpha }_{loss}$ and $\tau$. Notably, the kDU model showed poor reliability and agreement for ${\alpha }_{loss}$ for both MAP and hBayes.

**Table 7:** Reliability and agreement of the parameter estimates from the dual RL and dual RL kDU models in the reversal learning task for the three fitting procedures

	$\tau$		${\alpha }_{gain}$		${\alpha }_{loss}$		*κ*	
	*r*	ICC	*r*	ICC	*r*	ICC	*r*	ICC
MLE								
Dual RL	~.01	~.01 [.00/.99]	.11	.10 [.00/.90]	.40	.40 [.01/.59]	-	-
Dual RL kDU	~.01	~.01 [.00/.99]	~.01	.02 [.00/98]	.15	.16 [.00/.84]	.09	.07 [.33/.60]
MAP								
Dual RL	.53	.53 [.01/.46]	−.15	~.01 [.14/.85]	.40	.40 [.00/.60]	-	-
Dual RL kDU	.70	.71 [.00/.29]	.12	.12 [.00/.88]	.38	.38 [.00/.62]	.25	.25 [.00/.75]
hBayes								
Dual RL	.61	.61 [.01/38]	~.01	~.01 [.07/.93]	.56	.56 [.00/.44]	-	-
Dual RL kDU	.52	.53 [.00/.47]	−.09	~.01 [.07/.93]	.28	.28 [.06/.66]	−.07	~.01 [.38/.61]

Pearson correlations (*r*) and ICC(3,1). Values between square brackets indicate proportion of between-session variance and error variance

##### Internal validity

Again, we gauged the internal validity of the parameter estimates of the models by estimating how ${\alpha }_{gain}$ and the win-stay probability, and ${\alpha }_{loss}$ and the lose-shift probability relate using mixed-model beta-regression analyses. Across all fitting procedures, we found the expected positive relationship between ${\alpha }_{loss}$ and the lose-shift probability. For ${\alpha }_{gain}$ and the win-stay probability, this relation was robust for the dual-learning-rate model and the double-update model (see Table 8). Finally, similar to the bandit results, we found that although the MLE parameters showed low reliability, they did show significant internal validity.

**Table 8:** Results of beta-regression on learning rates and shifting strategies in the reversal learning task

	${\alpha }_{gain}$ *, win-stay*	${\alpha }_{loss}$*, lose-shift*
	$\beta$	$\beta$
MLE		
Dual RL	0.26 (0.03)***	0.51 (0.23)*
Dual RL kDU	0.20 (0.02)***	0.11 (0.04)*
MAP		
Dual RL	0.07 (0.06)*	0.59 (0.05)***
Dual RL kDU	0.25 (0.04)***	0.45 (0.06)***
hBayes		
Dual RL	0.27 (0.09)*	0.38 (0.06)***
Dual RL kDU	0.72 (0.28)***	0.38 (0.05)***

Standard errors are indicated between brackets; *** *p* < .001, * *p <* .05

##### Parameter identifiability

Similar to the bandit task, we performed parameter identifiability analyses on a representative set of 100 simulated participants for the reversal learning task, again focusing on the dual RL model and the dual RL kDU model.

In contrast to the bandit task, we found that all parameters showed good to excellent identifiability for the regularized fitting procedures, and even mostly good reliability for MLE (see Table 9). However, what stands out is the low identifiability of﻿ the ${\kappa}$ parameter, which may explain the low test–retest reliability in the real data. However, the levels of identifiability suggest that sufficient test–retest reliability is possible for most parameters when participants are stable over time. Indeed, our simulated participants showed a good level of reliability for ${\alpha }_{loss}$ and $\tau$, but, somewhat surprisingly, only moderate reliability for ${\alpha }_{gain}$ (see Table 10), and, as expected, poor reliability for *κ*.

**Table 9:** Simulated T1 identifiability of the parameter estimates from the dual RL and dual RL kDU models in the reversal learning task for the three fitting procedures

	$\tau$		${\alpha }_{gain}$		${\alpha }_{loss}$		*κ*	
	*r*	ICC	*r*	ICC	*r*	ICC	*r*	ICC
MLE								
Dual RL	.75	.64	.65	.65	.76	.76	-	-
Dual RL kDU	.77	.77	.79	.79	.89	.88	.07	.05
MAP								
Dual RL	.90	.89	.74	.72	.86	.78	-	-
Dual RL kDU	.77	.75	.81	.79	.89	.85	.08	.02
hBayes								
Dual RL	.88	.88	.74	.70	.82	.80	-	-
Dual RL kDU	.81	.75	.79	.79	.89	.88	~.01	~.01

Pearson correlations (*r*) and ICC(3,1)

**Table 10:** Simulated test–retest reliability of the parameter estimates from the dual RL and dual RL kDU models in the reversal learning task for the three fitting procedures

	$\tau$		${\alpha }_{gain}$		${\alpha }_{loss}$		*κ*	
	*r*	ICC	*r*	ICC	*r*	ICC	*r*	ICC
MLE								
Dual RL	.76	.76	.56	.56	.85	.85	-	-
Dual RL kDU	.60	.60	.62	.62	.70	.70	.20	.21
MAP								
Dual RL	.86	.85	.53	.53	.78	.79	-	-
Dual RL kDU	.68	.68	.67	.67	.82	.82	.56	.56
hBayes								
Dual RL	.90	.90	.59	.58	.83	.83	-	-
Dual RL kDU	.82	.82	.74	.72	.87	.84	.10	.09

Pearson correlations (*r*) and ICC(3,1)

##### Interim summary

Consistent with the findings for the bandit task, we found that regularizing methods (MAP and hBayes) improved parameter identifiability. Furthermore, for the assumed parameter space, and the given experimental design (number of trials, pairs, probabilities, etc.), this led to a sufficient level of identifiability for ${\alpha }_{loss}$ and $\tau$ to detect good reliability. The level of reliability was again lower for ${\alpha }_{gain}$ and poor for *κ*. We also see that ${\alpha }_{gain}$ was less strongly and less consistently correlated with shifting strategies. Most importantly, this was also reflected in the reliability measures, in which ${\alpha }_{gain}$ and *κ* performed very poorly across fitting procedures. On the other hand, the test–retest reliability for ${\alpha }_{loss}$ and $\tau$ in the empirical data reached moderate levels, an improvement over the bandit task.

#### Exploratory analyses: Modeling covariance

A recent paper suggests that joint modeling of the two time points (i.e., concurrent modeling of the data at T1 and T2), including a parameter that captures the correlation between the parameters across time points, substantially improves test–retest reliability in a reversal learning task (Waltmann et al., 2022). We therefore explored whether such joint modeling also improved test–retest reliability in our reversal learning data set and whether this held for the bandit task. To do so, we fitted joint hBayes models with and without a parameter for the correlation between parameters (${\alpha }_{gain},\;{\alpha }_{loss},\; \kappa , \;and\; \tau$) across time points to data from the two learning tasks and assessed their model fit. For brevity, these exploratory analyses focused on the best-fitting model according to the hBayes method: the RL kDU model.

For the bandit task, results showed improved model fit when correlations were estimated (DIC = 20,977) compared to when they were not (DIC = 21,002). However, surprisingly, there was no improvement in the test–retest reliability estimates compared to disjoint modeling (see Table 11).

**Table 11:** Reliability and agreement of the parameter estimates from the dual RL kDU model in the two tasks obtained using hBayes model fitting

		$\tau$		${\alpha }_{gain}$		${\alpha }_{loss}$		*κ*	
		*r*	ICC	*r*	ICC	*r*	ICC	*r*	ICC
Bandit	Disjoint	.46	.47	.18	.19	.27	.26	.25	.15
	Joint	.44	.43	.15	.15	.08	.03	.19	.18
Reversal	Disjoint	.52	.53	−.09	~.01	.28	.28	−.07	~0
	Joint	.59	.59	.02	.01	.37	.37	~.01	~.01

Pearson correlations (*r*) and ICC(3,1)

In contrast, for the reversal learning task, model fit did not improve when including correlations (DIC = 20,170 versus DIC = 20,164). However, for reversal learning, the joint modeling did increase the estimated reliability of the ${\alpha }_{loss}$ parameter for the dual RL kDU model, which however remained poor (see Table 11).

To address the question whether joint modeling could in principle improve estimates of test–retest reliability, we also ran these models on simulated data, which indicated that if the data sets for T1 and T2 were generated by the same set of parameters, it had excellent reliability (all *r*s > .98, see Table S6). However, if we consider parameter identifiability, the joint modeling approach did perform slightly worse.

In sum, although our simulation efforts are limited to the parameter values as we identified them in our data, and to a single model, our results suggest that joint modeling may provide a good estimate of the test–retest reliability, although this may be somewhat inflated, and it also may not be beneficial for parameter identifiability (cf., Waltmann et al., 2022, for more extensive analyses and comparison of this technique).

#### Exploratory analyses: Explaining variability with mood

Based on previous literature, we expected that current mood (Aylward et al., 2019; Bakic et al., 2014; Kube et al., 2023; Paul et al., 2020; Paul & Pourtois, 2017) could partly contribute to day-to-day variability in parameter estimates of the learning models. We tested this again using mixed-model beta-regression analyses across tasks and fitting procedures, using three factors (see Section "Mood" and Table S2): stress, wakefulness and happiness. For brevity, we report on the dual RL model only, but the exact same pattern of results was found for the double update version of the model (and no significant effects associated with the additional *κ* parameter). The results suggest that, if anything, mood impacts the learning rate for losses in the bandit task. More specifically, stress seemed to relate to an increased sensitivity to negative feedback, whereas happiness related to a decreased sensitivity to negative feedback. There is no evidence for any relationship with the reversal learning parameters (see Table 12). These results thus suggest that part of the within-participant variability in model parameters across time points can be explained by task-unrelated factors.

**Table 12:** Results of beta regression on learning rates and mood in the two tasks

	*Bandit task*		*Reversal learning task*	
	${\alpha }_{gain}$	${\alpha }_{loss}$	${\alpha }_{gain}$	${\alpha }_{loss}$
MLE				
Stress	−0.12 (0.10)	0.15 (0.11)	−0.14 (0.13)	−0.10 (0.14)
Wakefulness	−0.15 (0.10)	−0.13 (0.10)	−0.10 (0.15)	−0.11 (0.14)
Happiness	0.08 (0.10)	−0.16 (0.11)	0.01 (0.13)	−0.09 (0.14)
MAP				
Stress	−0.14 (0.08)	0.16 (0.07)*	−0.10 (0.11)	−0.21 (0.12)
Wakefulness	−0.15 (0.09)	−0.13 (0.07)	−0.10 (0.11)	0.14 (0.11)
Happiness	0.13 (0.08)	−0.17 (0.07)*	0.13 (0.10)	0.09 (0.12)
hBayes				
Stress	−0.11 (0.07)	0.19 (0.07)*	−0.05 (0.07)	−0.13 (0.04)
Wakefulness	−0.11 (0.07)	−0.15 (0.08)	0.01 (0.08)	0.02 (0.08)
Happiness	0.09 (0.08)	−0.19 (0.08)*	0.04 (0.07)	0.12 (0.09)

Standard errors are indicated between brackets; * *p* < .05

## Discussion

In this study, we assessed the test–retest reliability of two often-used learning tasks: a two-armed bandit task and a reversal learning task. We also included personality and cognitive ability measures to compare reliability between task measures and established stable traits. Behavioral task measures achieved moderate reliability, while personality and cognitive ability measures achieved high reliability. However, parameter estimates from reinforcement learning algorithms only achieved poor to moderate reliability, even though simulations indicated that our procedures could detect good reliability if participants were stable. Taking these results together, we conclude that participants’ learning parameters varied across time points. We discuss the potential implications and solutions for computational phenotyping and computational cognitive neuroscience.

As expected, our personality and cognitive ability measures showed high reliability. This corroborates previous findings showing generally good reliability for a short assessment of the Big Five factors of personality (with reliabilities ranging from .62 to .87; Donnellan et al., 2006) and for a short assessment of cognitive ability (Arthur Jr. et al., 1999; Arthur Jr. & Day, 1994; Bors & Forrin, 1995; with reliabilities ranging from .75 to .88). These measures thereby served as a benchmark for the reliability of behavioral and model-based task measures. Our mood measure mostly showed moderate reliability, suggesting relatively stable mood across the five weeks. This is in line with previous research showing that positive and negative affect were moderately stable over a two-month period, with reliabilities ranging from .59 to .71 (Watson & Clark, 1994).

A prerequisite of test–retest reliability of model parameters is parameter identifiability. That is, if parameters cannot be identified adequately at a single time point, this induces measurement noise, subsequently harming reliability (Zorowitz & Niv, 2022). Therefore, as a first step, we performed a simulation study, based on the empirical parameter distributions, in which we showed that the combination of our computational models and experimental tasks can result in moderate to good parameter identifiability. This indicates that our procedures, which we believe reflect the most common approach in the field, can detect stable learning parameters reasonably well. Given that we established sufficient identifiability to find good test–retest reliability when participants showed stable behavior, we concluded that the poor to moderate test–retest reliability of reinforcement learning parameters suggests that participants’ learning strategies were unstable. This corroborates previous findings in a reversal learning task (Waltmann et al., 2022) showing poor to moderate test–retest reliability of learning rates (.16 to .59 as compared to −.09 to .56 in our sample) and inverse temperatures (−.03 to .64 as compared to .01 to .71 in our sample). Moreover, our results extend previous findings in a bandit task showing moderate reliability of behavioral measures (Pratt et al., 2021), to even worse reliability of reinforcement learning parameters in this task.

What could underlie this instability? A first possibility is the stochastic nature of the task. Specifically, the stochastic nature of the feedback in both tasks and the choice-dependent reversal rule in the reversal learning task may have affected task dynamics. However, stochasticity only puts a general limit on the reliability, which is also captured by our simulations. Stochasticity thus fails to explain the difference between the simulated and empirical reliability results.

A second possibility is that participants truly differed across time points, due to either trait-like or state-like factors. Previous research showed that, for example, traits such as attention-deficit/hyperactivity disorder (ADHD; Hauser et al., 2016; Kofler et al., 2013; Salum et al., 2019) and states such as attentiveness (Aristodemou et al., 2022) are associated with increased variability across time points. Although beyond the scope of this paper, it is important that future studies investigate how such traits and states previously shown to relate to increased behavioral variability relate to variability in reinforcement learning parameters across time points. In particular for traits and states that are (part of) the problem one wishes to characterize, because failing to acknowledge this variability can lead to different—and more importantly, incorrect—characterizations of individuals at different time points. In light of this, exploratory results showed that part of the within-participant variability in our data could be explained by the participants' mood. This suggests that day-to-day fluctuations in task-unrelated factors should be considered to adequately uncover learning strategies. It also suggests that variability itself may be a variable of interest for establishing computational phenotypes. For example, variability in mood together with learning rates may provide valuable information for diagnosing and monitoring depression (Chase et al., 2010; Kube et al., 2023) and bipolar disorder (Holmes et al., 2011; Pratt et al., 2021).

Another possibility is that the variance that we measure reflects variability in learning strategies rather than variability in learning parameters. Although the canonical approach in computational psychiatry is to perform model selection based on population-level data (which is in fact the only way to make group comparisons in learning parameters themselves), other research focuses on finding the best-fitting model on the participant level. For instance, common heuristic models for decision-making have no free parameters, and research is focused on strategy distributions across populations (Mata et al., 2015; Scheibehenne et al., 2013). Indeed, an increasing number of studies suggest there are large individual differences in learning strategies (Lee & Webb, 2005; Zadelaar et al., 2019). More importantly, some even suggest that strategies are changing within participants throughout learning tasks (Rieskamp & Otto, 2006; Scheibehenne et al., 2013). For example, in the context of the bandit task, it may well be that participants stop learning and simply start exploiting one of the options after a number of trials (thus reducing the learning rate to zero). Our approach, in which we determined the best-fitting model across participants, only allowed for quantitative individual differences (i.e., in learning parameters), not qualitative differences (i.e., in learning strategies). Future studies are advised to estimate individual learning strategies, for example, by using mixture modeling (e.g., Bartlema et al., 2014; Schaaf et al., 2019), to assess how stable these strategies are across time points, and whether they change during the task.

Both these alternative views on sources of variance imply that parameter estimates obtained at a single time point are unreliable measures to characterize an individual. This complicates the straightforward implementation of cognitive phenotyping. That is, our results suggest that phenotyping is unreliable with canonical RL tasks and the amount of data commonly available. Here we discuss a few ways to save the phenotyping approach. First, identifiability could be further improved, for example by increasing the number of administered trials (e.g., Shahar et al., 2019) or by jointly modeling multiple data sources (e.g., response times or neural data; Ballard & McClure, 2019; Fontanesi et al., 2019; Miletić et al., 2021; Pedersen et al., 2017; Shahar et al., 2019; Turner et al., 2013, 2016). Arguably, one of the easiest methods to increase reliability is to collect more data for each time point (e.g., Rouder & Haaf, 2019). However, this method is limited, especially in clinical populations, due to potential fatigue (Zorowitz & Niv, 2022), and would not fully address the variability between time points. Taking variability seriously, one could start collecting data across multiple time points to better distinguish stability and variability in task behavior (for example using dynamic structural equation modeling; Aristodemou et al., 2022; Asparouhov et al., 2018). However, this method may be unpractical and requires larger sample sizes due to increased drop-out rates. Another method is to further investigate the sources of variability cross-sectionally (e.g., effects of mood) and to incorporate them in reinforcement learning algorithms.

In sum, based on our results, we conclude that current common collection and computational modeling procedures are insufficiently reliable for cognitive phenotyping on the individual level. Importantly, it is insufficient to show that, based on simulations alone, parameter identifiability is good or excellent. As we have demonstrated here, good results in simulations only show that parameter identifiability is potentially good, which is a minimum requirement, but we also show that this does not guarantee that participants are stable over time. Thus, besides showing that models can be recovered well and that parameters can be identified, for computational phenotyping to work it is also necessary that parameters are stable within participants over multiple time points. This necessarily involves longitudinal data collection.

On a more positive note, insufficient or unproven test–retest reliability does not directly imply that one cannot reliably assess group differences (e.g., clinical versus control). If groups differ enough in their mean parameter values, even with low test–retest reliability, one can reliably detect these differences (Haines et al., 2020; Hedge et al., 2018). Also, high identifiability suggests that individual parameters are adequately modeled at the current moment and thus that variables that are concurrently measured can be related to these parameters. Accordingly, computational neuroimaging studies can still provide insight into the neural correlates of reinforcement learning processes. As such, we do not believe that the current results, which we intuit generalize to other similar tasks and computational models, discredit most previous work in computational cognitive neuroscience and psychiatry (see also Palminteri & Chevallier, 2018), but rather emphasizes the importance and potential value of taking variability seriously.

One of our secondary aims was to assess how model complexity affects test–retest reliability. Results from simulations, in which we investigated reliability in a dual RL model and a more complex dual RL kDU model, were mixed. In the bandit task, results somewhat consistently showed higher reliability for *α*_*gain*_ and *α*_*loss*_ in the dual RL model than in the more complex dual RL kDU model. However, results showed similar reliability for *τ* across models. In the reversal learning task, results consistently showed lower reliability for *α*_*gain*_ and higher reliability for *τ* in the simpler model; results for *α*_*loss*_ were very inconsistent. Based on these mixed results in combination with our finding that the identifiability of the *κ* parameter in the dual RL kDU model was generally low, we believe it is inappropriate to draw any conclusions on the effect of model complexity on test–retest reliability.

## Conclusion

In conclusion, reinforcement learning algorithms can be used to characterize the processes underlying learning. However, often-used learning tasks and computational models are unsuitable to draw conclusions on an individual level, and cannot, as yet, be used as an alternative for individual diagnosis of psychiatric disorders. In order to improve diagnosis and treatment, variability should be taken seriously and incorporated in computational models of learning.

In the current paper we have focused on canonical tasks and a common set of computational models to analyze them. We believe that these results will generalize to other dynamic learning tasks and different families of models, but this should be empirically established. This is beyond the scope of the current paper, but we hope to motivate researchers to move beyond simple identifiability measures and establish the test–retest reliability of their tasks and models (see also Parsons et al., 2019). We also invite them to use our dataset if they believe they have analytical tools at their disposal that may allow for better estimates that result in higher reliability (https://osf.io/pe23t/).

### Supplementary Information

Below is the link to the electronic supplementary material.

Supplementary file1 (DOCX 39 KB)

Supplementary file2 (DOCX 3918 KB)

## Data Availability

Data and materials are freely available at https://osf.io/pe23t/.

## Footnotes

1 Running the identifiability analyses on the T2 data set or a joint T1 and T2 data set gave similar results.

## Code availability

Analysis code is freely available at https://osf.io/pe23t/.
