# Supplementary material for: Test–retest reliability of reinforcement learning parameters
Source: Behav Res Methods. 2023 Sep 8;56(5):4582–99. doi: 10.3758/s13428-023-02203-4 (PMC11289054; doi:10.3758/s13428-023-02203-4)
Supplement: Supplementary file 1 — Supplementary file1 (DOCX 39 KB) [file 13428_2023_2203_MOESM1_ESM.docx]

**Supplemental Materials**

**Table S1.** *Reliability and Agreement for Personality, Cognitive Measures and Mood*

| IPIP subscales | Pearson correlation | ICC(3,1) |
| --- | --- | --- |
| Extraversion | .90 | .88 [.01/.11] |
| Agreeableness | .78 | .78 [.00/.21] |
| Conscientiousness | .79 | .79 [.00/.21] |
| Neuroticism | .84 | .84 [.01/.11] |
| Openness | .74 | .74 [.01/.25] |

*ICC[between session variance/residual variance]*

| Cognitive | Pearson correlation | ICC(3,1) |
| --- | --- | --- |
| Raven | .60 | .60 [.14/.26] |

| Moods | Pearson correlation | ICC(3,1) |
| --- | --- | --- |
| Tired | .74 | .74 [.02/.24] |
| Happy | .74 | .74 [.00/.26] |
| Hungry | .38 | .38 [.00/.62] |
| Stressed | .71 | .71 [.00/.29] |
| Awake | .52 | .52 [.00/.48] |
| Worried | .68 | .68 [.00/.32] |
| Bored | .65 | .60 [.07/.33] |
| Relaxed | .77 | .77 [.00/.23] |

**Table S2.** *Exploratory Factor Analyses on Mood Measure*

|  | Factor1  (stress) | Factor2  (wakefulness) | Factor3  (happiness) |
| --- | --- | --- | --- |
| stressed | 0.93 |  |  |
| worried | 0.92 |  |  |
| tired |  | -0.7 |  |
| awake |  | 1.01 |  |
| happy |  |  | 0.96 |
| hungry | 0.5 |  |  |
| bored |  |  |  |
| relaxed |  | 0.35 |  |

We performed a maximum likelihood exploratory factor analyses with three factors and with oblique rotation to reduce dimensionality in the mood data. This resulted in the following factor structure: stress, wakefulness and happiness.

**Parameter distributions and priors**

To construct the priors for the maximum a posteriori estimation (MAP) we have used the parameter distributions as estimated with MLE for the sessions in T1 and T2 (see Table S1 and S2). Given that yesterday’s posterior is todays prior we have compared model fits at T2 using the prior based on distribution of either T1 and T2 (a special case of empirical priors; cf., Gershman, 2016). We have implemented directly with MAP. In the hBayes framework this results in either providing T1 distributions as priors for the hyper parameters, or using the standard method with uninformed priors. Interestingly, comparing model fits suggests that this using either T1 or T2 data in parameter estimates does not have a significant impact on model fit or selection (see Table 1). This may not be surprising given that indeed we use priors based on the same population measured at different time points. As can be expected comparing parameter estimates also showed that these two methods led to extremely similar parameter estimates (all Pearson *r’*s > .92).

**Table S3.** *Parameter distribution based on MLE fitting used for* *MAP fitting*
**Bandit (MLE)**

|  | *α* | | *α_gain_* | | | | *α_loss_* | | *κ* | | *τ* | |
| --- | --- | --- | --- | --- | --- | --- | --- | --- | --- | --- | --- | --- |
|  | T1 | T2 | | T1 | T2 | T1 | | T2 | T1 | T2 | T1 | T2 |
| Simple RL | .40  (.27) | .41  (.27) | | - | - | - | | - | - | - | 5.36  (2.99) | 5.29  (2.99) |
| Simple RL DU | .19  (.22) | .14  (.17) | | - | - | - | | - | - | - | 5.37  (3.55) | 6.45  (3.57) |
| Simple RL kDU | .35  (.27) | .30  (.27) | | - | - | - | | - | .15  (.29) | .20  (.32) | 5.90  (3.15) | 6.78  (3.16) |
| Dual RL | - | - | | .68  (.34) | .61  (.36) | .26  (.24) | | .28  (.25) | - | - | 5.37  (2.92) | 5.59  (3.03) |
| Dual RL DU | - | - | | .28  (.30) | .20  (.21) | .15  (.23) | | .10  (.18) | - | - | 6.62  (3.36) | 7.39  (3.23) |
| Dual RL kDU | - | - | | .65  (.35) | .56  (.39) | .22  (.24) | | .19  (.23) | .21  (.31) | .26  (.36) | 6.22  (3.17) | 6.88  (3.17) |

**Reversal Learning (MLE)**

|  | *α* | | *α_gain_* | | | | *α_loss_* | | *κ* | | *τ* | |
| --- | --- | --- | --- | --- | --- | --- | --- | --- | --- | --- | --- | --- |
|  | T1 | T2 | | T1 | T2 | T1 | | T2 | T1 | T2 | T1 | T2 |
| Simple RL | .66  (.34) | .71  (.31) | | - | - | - | | - | - | - | 2.56  (2.59) | 2.13  (2.16) |
| Simple RL DU | .54  (.30) | .54  (.30) | | - | - | - | | - | - | - | 1.05  (.61) | .98  (.58) |
| Simple RL kDU | .50  (.30) | .53  (.29) | | - | - | - | | - | .36  (.37) | .39  (.37) | 1.75  (1.07) | 1.72  (1.16) |
| Dual RL | - | - | | .63  (.37) | .70  (.34) | .69  (.30) | | .67  (.33) | - | - | 2.81  (2.12) | 2.79  (2.51) |
| Dual RL DU | - | - | | .35  (.26) | .69  (.34) | .38  (.31) | | .67  (.33) | - | - | 3.39  (2.92) | 2.82  (2.59) |
| Dual RL kDU | - | - | | .65  (.38) | .69  (.34) | .63  (.31) | | .67  (.33) | .19  (.24) | .47  (.30) | 2.85  (2.21) | 2.82  (2.59) |
| Mean(standard deviation) of parameter estimates based on MLE | | | | | | | | | | | | |

**Table S4.** *Correlations between parameter estimates obtained with MAP and hBayes fitting procedures for the bandit task*

| *Dual RL* | | | |  |
| --- | --- | --- | --- | --- |
| **MAP/hBayes** | *τ* | *α_gain_* | *α_loss_* |  |
| *τ* | .99** |  |  |  |
| *α_gain_* |  | .94** |  |  |
| *α_loss_* |  |  | .99** |  |
| *Dual RL kDU* | | | | |
| **MAP/hBayes** | *τ* | *α_gain_* | *α_loss_* | *κ* |
| *τ* | .91** |  |  |  |
| *α_gain_* |  | .71** |  |  |
| *α_loss_* |  |  | .79** |  |
| *κ* |  |  |  | .32** |

**Table S5.** *Correlations between parameter estimates obtained with MAP and hBayes fitting procedures for the reversal learning task*

| *Dual RL* | | | |  |
| --- | --- | --- | --- | --- |
| **MAP/hBayes** | *τ* | *α_gain_* | *α_loss_* |  |
| *τ* | .95** |  |  |  |
| *α_gain_* |  | .86** |  |  |
| *α_loss_* |  |  | . 85** |  |
| *Dual RL kDU* | | | | |
| **MAP/hBayes** | *τ* | *α_gain_* | *α_loss_* | *κ* |
| *τ* | . 87** |  |  |  |
| *α_gain_* |  | .54** |  |  |
| *α_loss_* |  |  | .68** |  |
| *κ* |  |  |  | .51** |

**Reliability of joint hBayes modeling**

In the first step, as before, we used two data sets that were based on the same set of underlying parameters that were representative for the estimated parameters of the subject in our studies. However, we identified that the model failed to converge due to a very low average *κ* parameter, therefore we have increased the value of the parameter for these analyses (mean *κ* in population and previous simulations was .2, in these simulations .5). Doing so did not only help the model converge, but also showed that a higher value of this parameter increases it identifiability and reliability overall. First, we considered the identifiability by looking at parameter recovery, which showed us that the identifiability, although generally good, actually suffered a bit in comparison to the identifiability we have reported for in the main manuscript (see Table 4 and Table 9). However, less surprisingly, and in line with Waltmann and colleagues (2022), the joint modeling yielded very high measures of reliability.

**Table S6.** *Identifiability and* *T1-T2 reliability of simulated dual RL kDU data*

| **Identifiability** | *τ* | | | *α_gain_* | | | | | *α_loss_* | | | | | *κ* | | | |  |
| --- | --- | --- | --- | --- | --- | --- | --- | --- | --- | --- | --- | --- | --- | --- | --- | --- | --- | --- |
|  | r | ICC | | | r | | ICC | | | r | | ICC | | r | | ICC | |  |
| Bandit | .70 | | .67 | | | .55 | | .12 | | | .67 | | .66 | | .36 | | .33 | |
| RevL | .70 | | .69 | | | .80 | | .71 | | | .74 | | .74 | | .77 | | .74 | |

| **Test-retest** | *τ* | | | *α_gain_* | | | | | *α_loss_* | | | | | *κ* | | | |  |
| --- | --- | --- | --- | --- | --- | --- | --- | --- | --- | --- | --- | --- | --- | --- | --- | --- | --- | --- |
|  | r | ICC | | | r | | ICC | | | r | | ICC | | r | | ICC | |  |
| Bandit | .99 | | .99 | | | .97 | | .96 | | | .99 | | .99 | | .98 | | .98 | |
| RevL | .99 | | .99 | | | .99 | | .99 | | | .99 | | .99 | | .99 | | .99 | |
| *Note.* Pearson correlations and ICC(3,1). All between session variance < .01. | | | | | | | | | | | | | | |  | |  | |

**Table S7.** *Correlations between Shifting Strategies and Mood in the Two Tasks*

|  | *Bandit Task* | | *Reversal Learning Task* | |
| --- | --- | --- | --- | --- |
|  | *Win-stay* | *Lose-shift* | *Win-stay* | *Lose-shift* |
|  | | |  |  |
| stress | -.05 (.08) | .02 (.05) | -.19 (.13) | -.18 (.06)* |
| wakefulness | -.03 (.06) | -.02 (.05) | -.17 (.12) | .04 (.05) |
| happiness | -.01 (.08) | -.01 (.06) | .01 (.14) | .06 (.07) |
| *Note.* Standard errors are indicated between brackets; * *p* < .05. | | | | |

**Details on excluded participants**

Exclusion criteria were based on a pilot study. These criteria were, in this specific order: (1) failing to provide a valid Amazon Mechanical Turk ID, (2) failing the comprehension checks or indicating comprehension problems at the end of the experiment, (3) always choosing the stimulus on one side of the screen, (4) less than 50% correct responses, (5) timed-out responses on more than 20% of trials, and (6) not completing the full experiment. The number of excluded participants based on these six criteria for each of the two tasks is presented in Table S8. To keep the conditions as similar as possible for the two tasks, we stuck to the same exclusion criteria in both tasks. We used 20% timed-out responses as cutoff because missing more than this amount of trials would mean participants would miss over 50 trials in the reversal learning task. This in turn would lead to too few reversals to be able to investigate the effect of these events. In addition, failing to respond within 5 seconds (while most participants respond within 2 seconds) likely indicates inattention, which would lead to poor data quality and in turn to underestimation of the test-retest reliability. Note, however, that after excluding participants based on laterality and correct responses, only very few participants were excluded based on timed-out responses.

**Table S8.** *Number of Excluded Participants per Task and Timepoint*

|  | *Bandit Task* | | | *Reversal Task* | |
| --- | --- | --- | --- | --- | --- |
|  | *T1* | *T2* | *T1* | | *T2* |
|  | | | |  | |
| (1) MTurk ID | 2 | 4 | 2 | | 4 |
| (2) Comprehension | 3 | 0 | 0 | | 0 |
| (3) Laterality | 6 | 5 | 14 | | 9 |
| (4) Correct responses | 14 | 8 | 40 | | 4 |
| (5) Time-outs | 2 | 2 | 5 | | 0 |
| (6) Incomplete | 0 | 5 | 2 | | 0 |
| Total | 27 | 24 | 63 | | 17 |
