# Supplementary material for: Test–retest reliability of reinforcement learning parameters
Source: Behav Res Methods. 2023 Sep 8;56(5):4582–99. doi: 10.3758/s13428-023-02203-4 (PMC11289054; doi:10.3758/s13428-023-02203-4)
Supplement: Supplementary file 2 — Supplementary file2 (DOCX 3918 KB) [file 13428_2023_2203_MOESM2_ESM.docx]

**Experimental materials**

Below we show the experimental screens as seen by the participants. We first show the Reinforcement Learning Experiment and then the Reversal Learning Experiment. Participants completed only one of the two experiments. For each experiment, there were two sessions (T1 and T2). The screens for these sessions were identical.

On-screen buttons are shown [between square brackets]. These directed the participants to the next screen. Below, we separate the experimental screens with a “===” sign on the left hand side.

| Where applicable, we add comments in a grey box like this. |
| --- |

**1. REINFORCEMENT LEARNING EXPERIMENT**

===

# **Welcome!**

In this experiment you will have to make a series of decisions.

In each trial, you have to choose between **two different objects.**

One of the objects is always more valuable than the other. It is your task to learn **which objects are more valuable.**

Your goal is to **maximize** the **total** amount of money that you can win.

Click continue to proceed.

[Continue]

===

A choice can have three outcomes: either the chance to **gain 1$**, or **lose 1$**, or a **neutral (0$)** outcome where you neither lose nor win.

If your response is **too late (0$)** you neither win nor lose.

After each choice, you will see your outcome during that round. This is the bonus you could earn in this round if this round is selected.

**Attention: You do not receive the outcomes for every round you play.**

Instead, your bonus for this task will be calculated as follows:
Once you have completed this HIT, the computer will **randomly select 3 rounds you played**. It will add up the results you had in this round: either you won (+1$), or you lost (-1$), the outcome was neutral (0$) or you were too late (0$).

So **your maximum bonus during this HIT is 3$**. Your bonus cannot become negative.

The entire HIT consists of **four games** with **40 rounds** each.

Click on the button to do two practice trials.

[Continue]

===


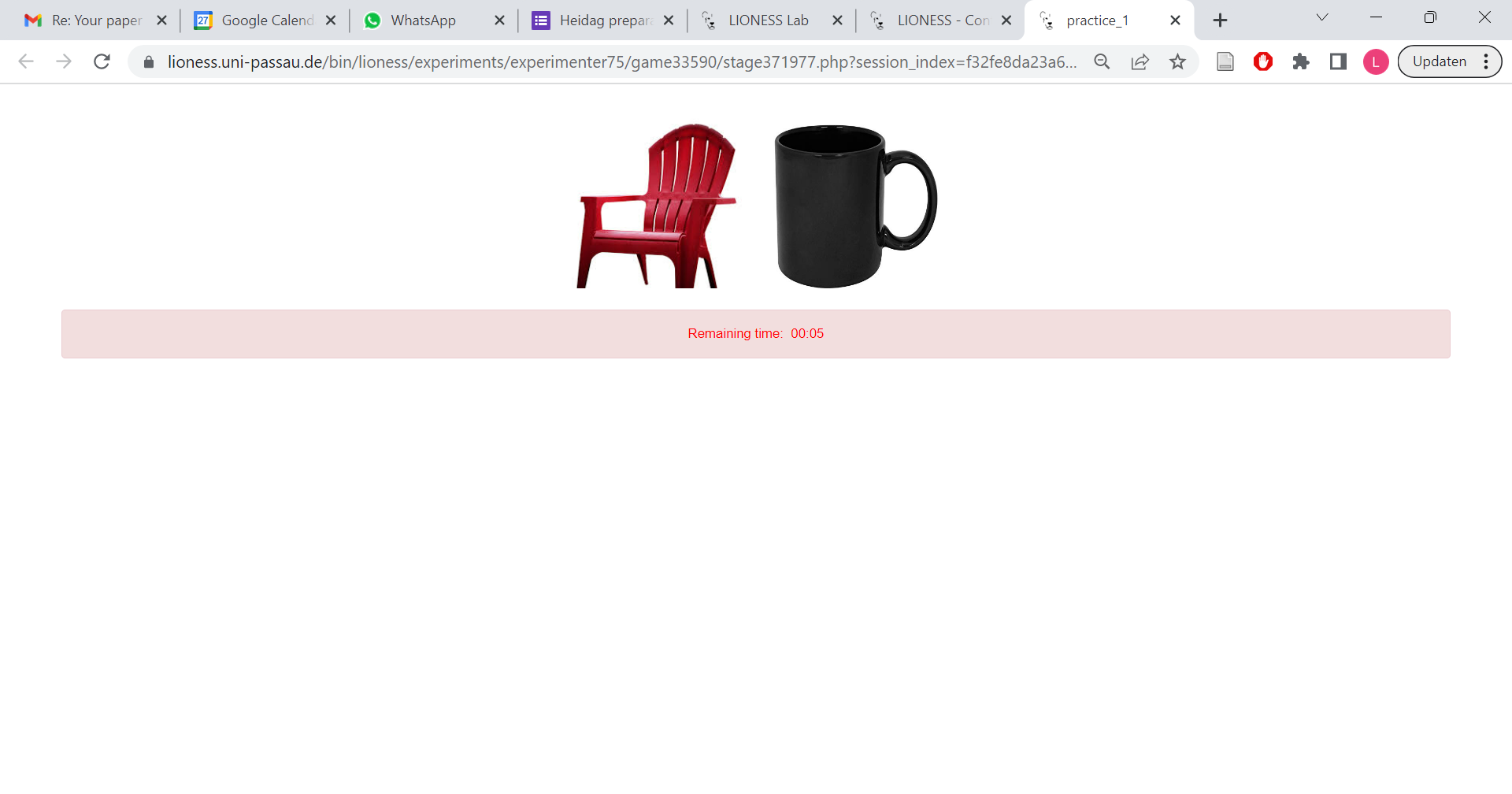


| This **decision screen** was shown for a *maximum of 5 seconds*. When the participant chose one of the options, a thick purple border appeared around the selected option, and the participant was directed to the feedback screen. If a participant had not made their choice yet after 5 seconds, they received a feedback screen reading ‘too late’ (see below). At the bottom of this description of the instructions, we show the different stimulus objects we used. |
| --- |

===


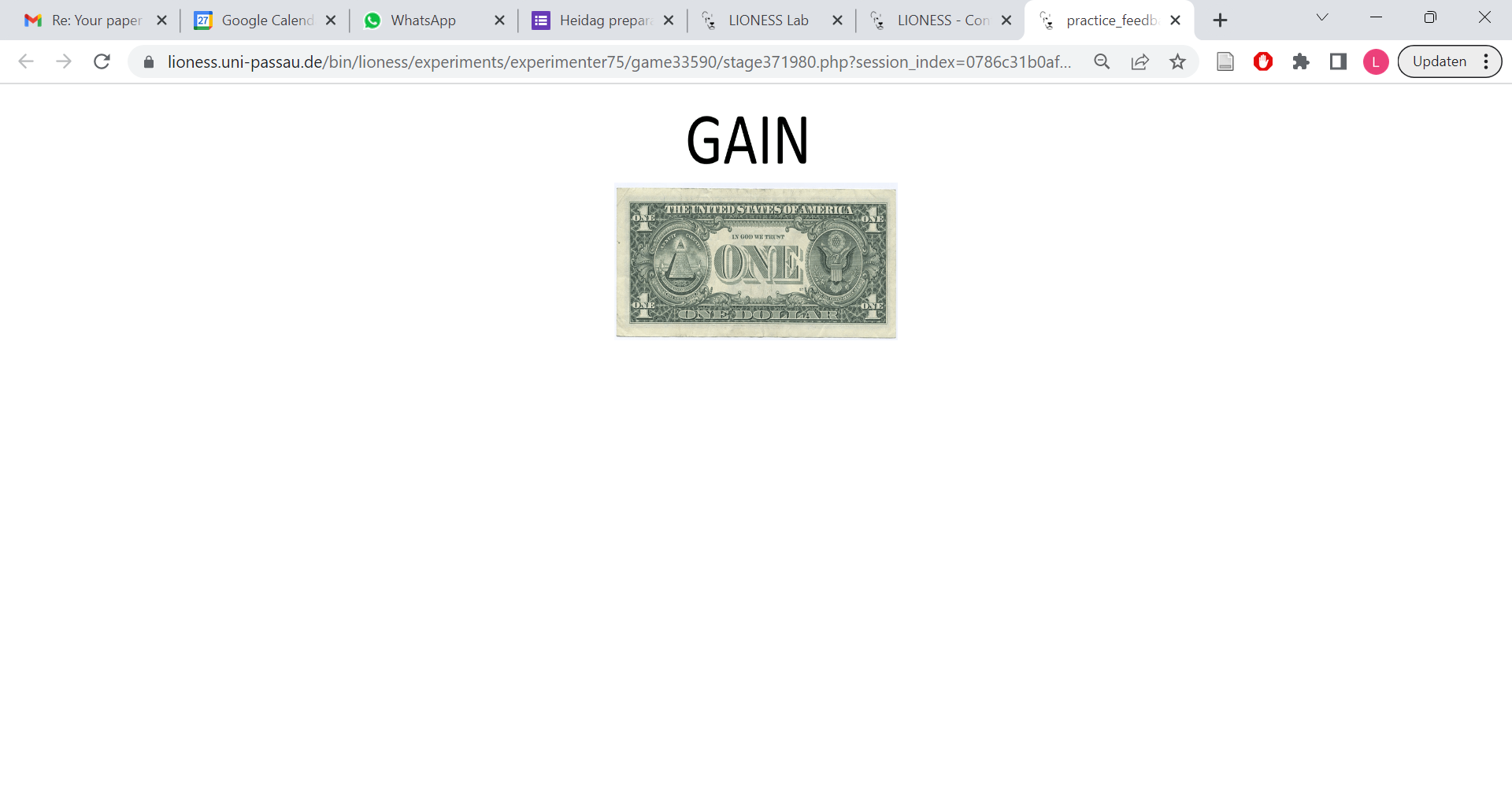

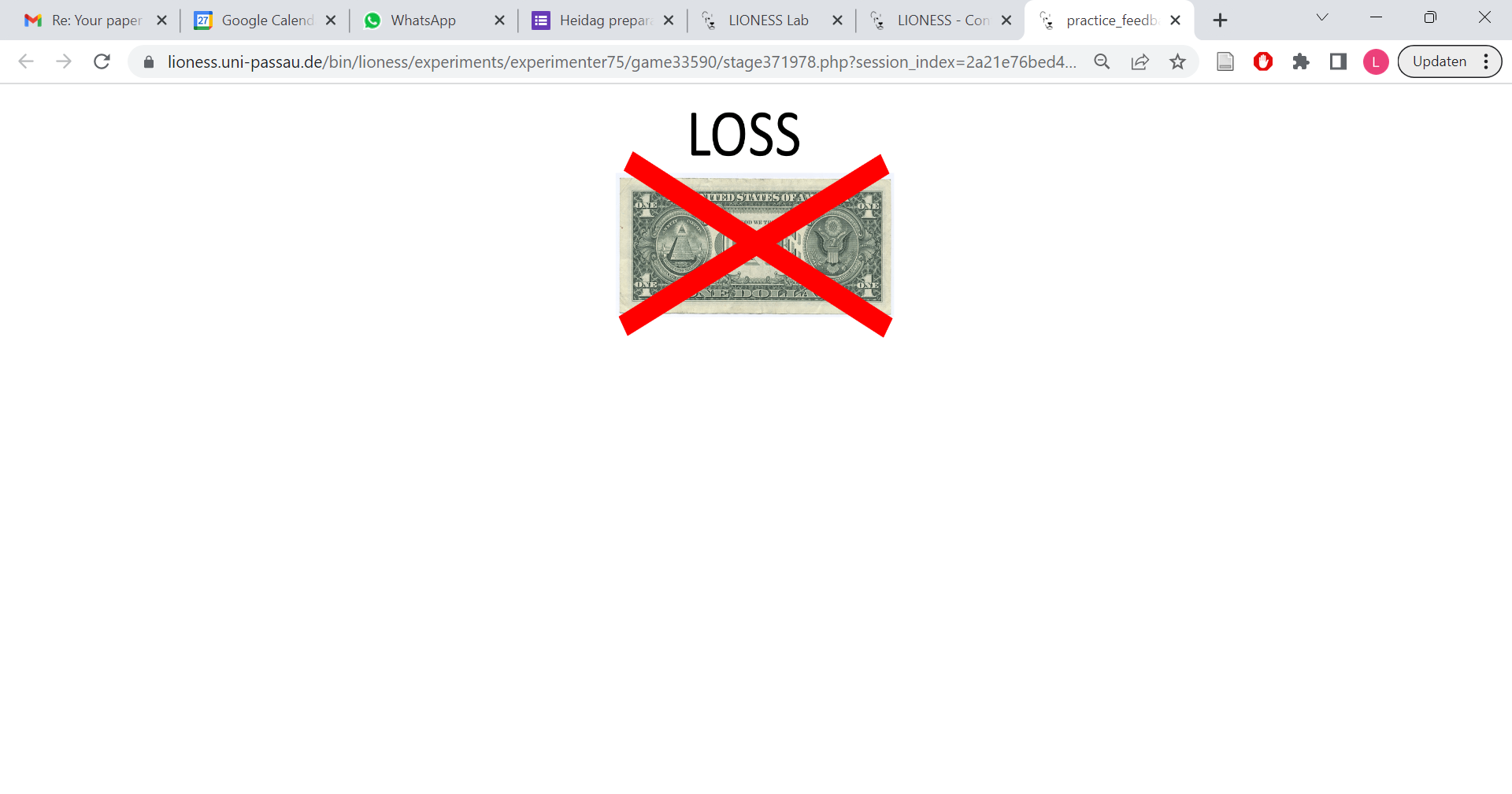

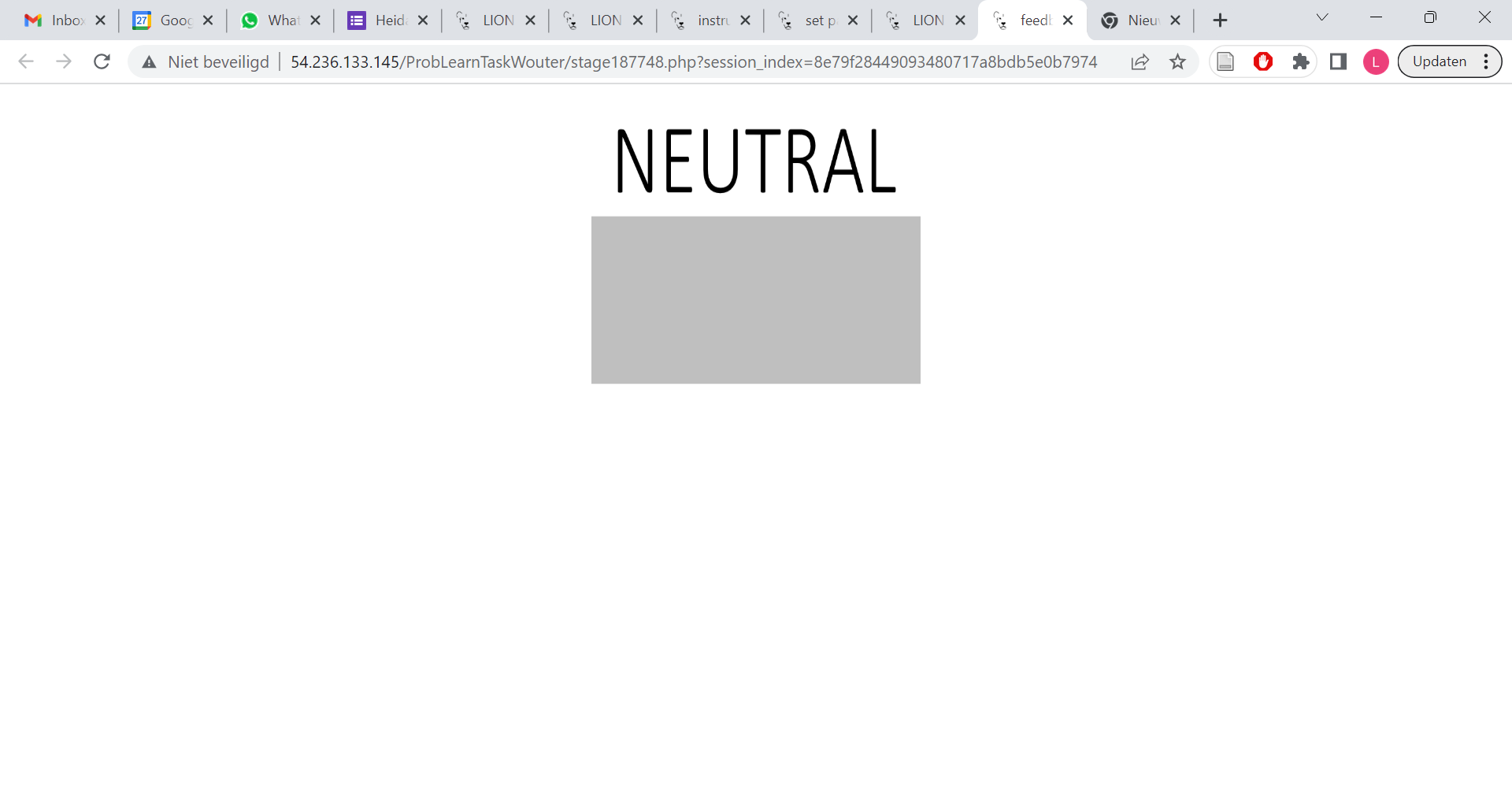

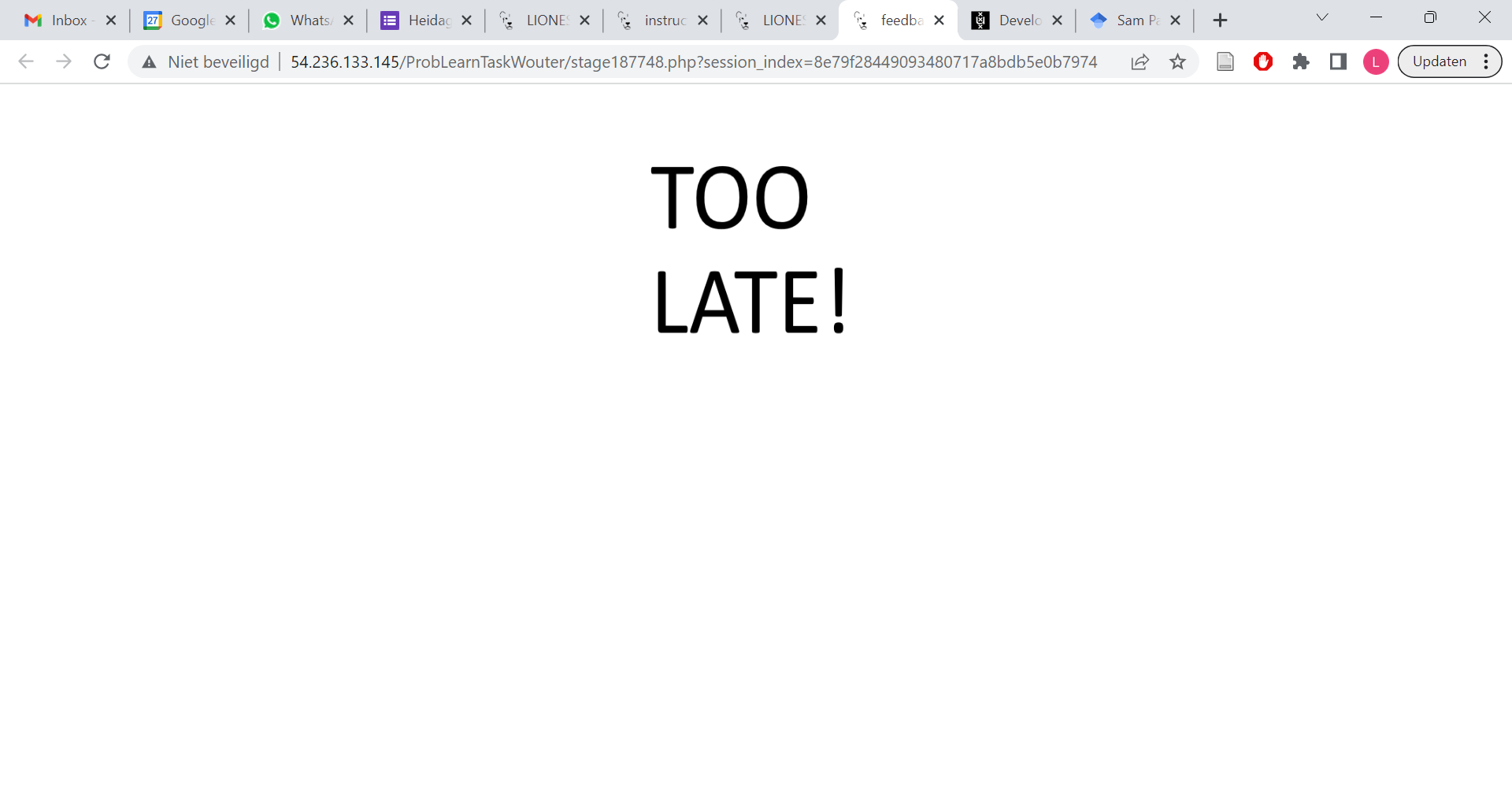


| This **feedback screen** was shown *for 2 seconds*. In each trial, it showed one of these four images, dependent on the outcome. |
| --- |

| After 2 practice trials, the experiment itself started. |
| --- |

===

# **You're ready**

Click continue to start the **first game.**

In this game there will be 40 rounds.

[continue]

===

| 40 trials ensued. Each trial consisted of a decision screen and a feedback screen (see above). |
| --- |

# **This is the end of this round.**

Click continue to start the **next round** of the experiment.

In the next round you will play the same game, but with different images. There will again be 40 rounds.

[Next block]

| 40 trials ensued with a fresh set of stimuli. As before, each trial consisted of a decision screen and a feedback screen (see above). |
| --- |

===

# **This is the end of this round.**

Click continue to start the **next round** of the experiment.

In the next round you will play the same game, but with different images. There will again be 40 rounds.

| 40 trials ensued with a fresh set of stimuli. As before, each trial consisted of a decision screen and a feedback screen (see above). |
| --- |

===

# **This is the end of this round.**

Click continue to start the **next round** of the experiment.

In the next round you will play the same game, but with different images. There will again be 40 rounds.

[continue]

| 40 trials ensued with a fresh set of stimuli. As before, each trial consisted of a decision screen and a feedback screen (see above). |
| --- |

===

### **Bonus earnings**

Your earnings are calculated by randomly selecting 3 rounds from the HIT and adding the results you earned during those rounds.

Round 25, 46 and 70 were selected. In those rounds, you collected a total of **Points**.

These Points are worth **$XXX**. This is your bonus for this HIT.
Note that any bonus you earn will be paid on top of your guaranteed participation fee of **$3.00**.

Next, we would like to ask you a few questions and do two more tests before you complete this HIT.

[Continue]

===

We would like to hear about your experience with this experiment. Please let us know whether you had any problems!

Did you understand the task?

Not at all 0 0 0 0 0 Yes, absolutely

If it was not easy to understand the task or if you experienced any problems, please describe **what aspects of the task you were unsure about**:

[open text]

What was your **strategy** to solve this HIT?

[open text]

===

| After completing the reinforcement learning task, the participants were directed to a questionnaire, starting with Raven matrices. |
| --- |

# **Instructions**

Welcome! In this test you will have to make a series of decisions.

At the top of the screen you will see a **series of symbols**. At the bottom, you will see individual symbols.

Your task is to look at the series at the top and find out what the missing part must look like to complete the pattern correctly, both along the rows and the columns.

Pick the correct solution out of the options at the bottom. Each round has only one correct answer. **For each correct answer you provide, you win 10 cents!**

This test has 9 rounds. Click continue to begin.

[Continue]


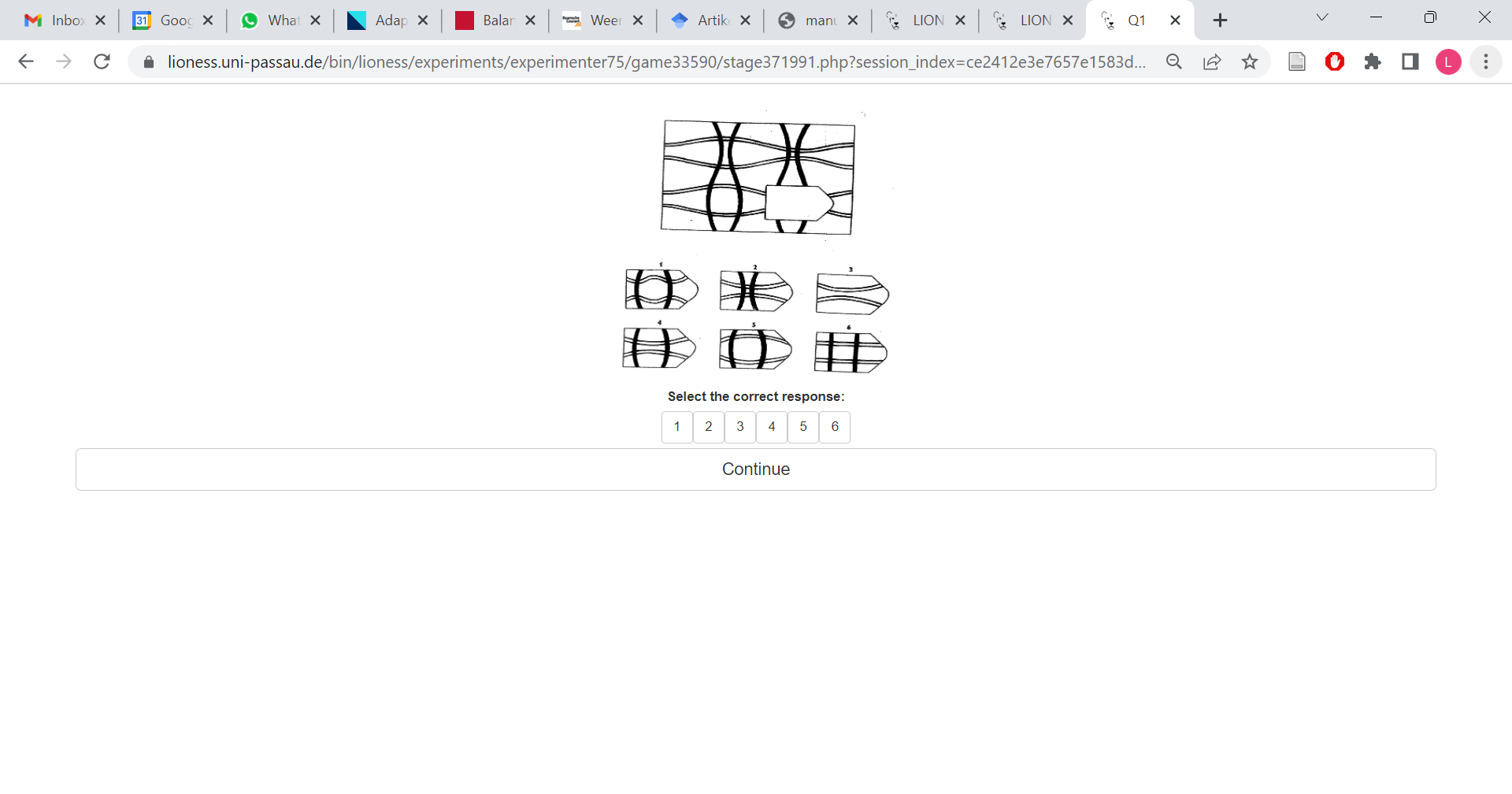


===


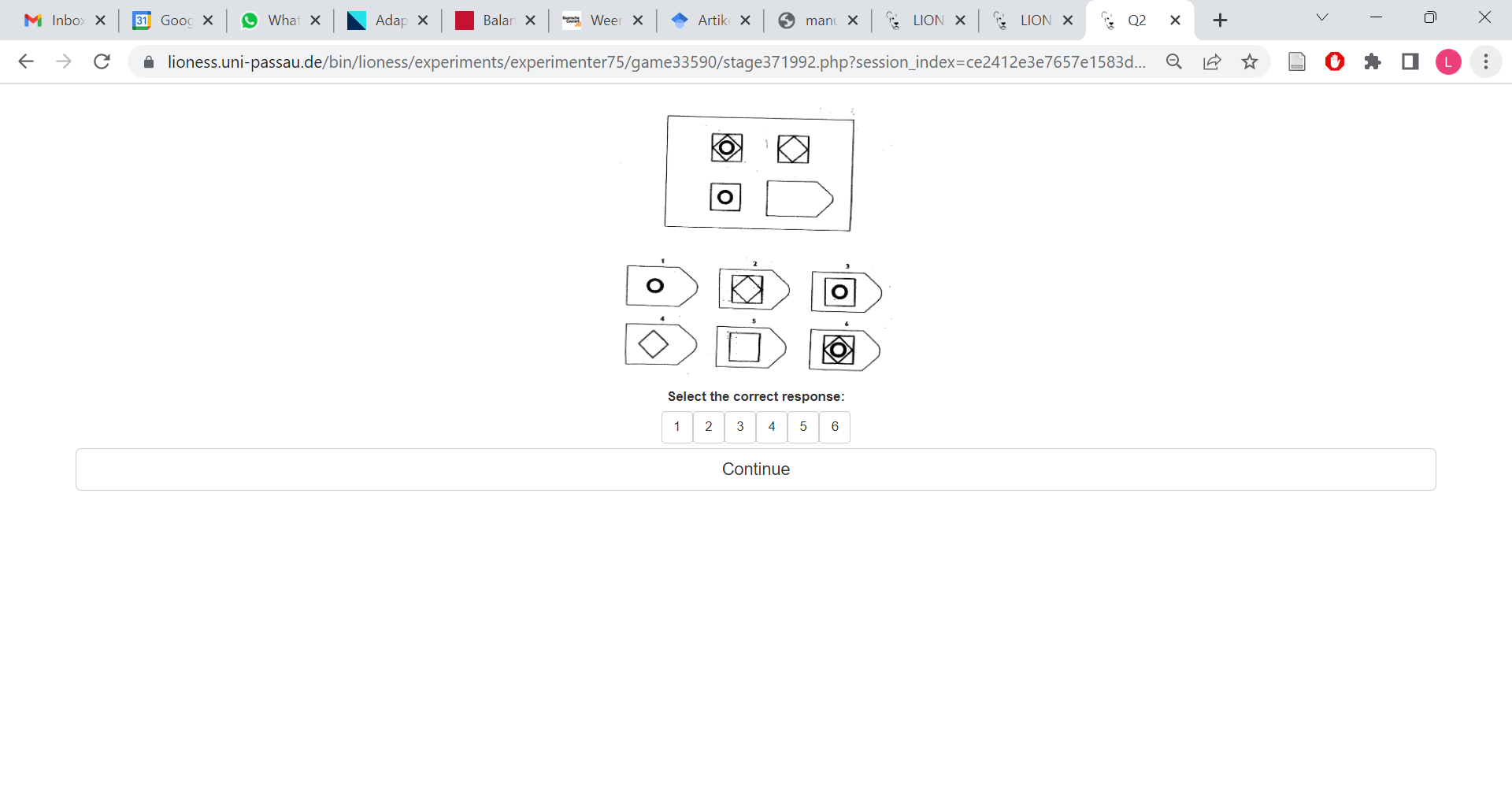


===


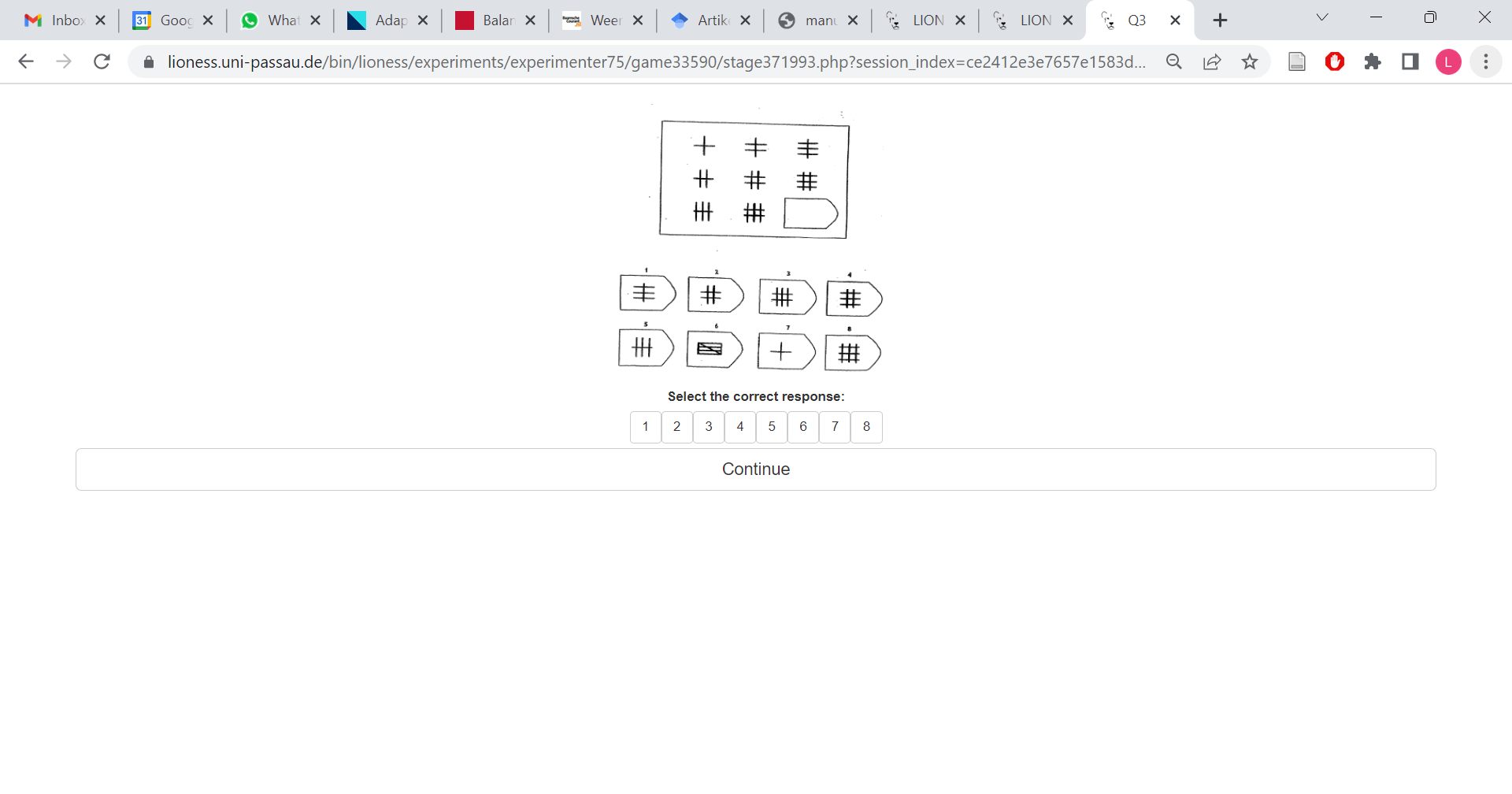


===


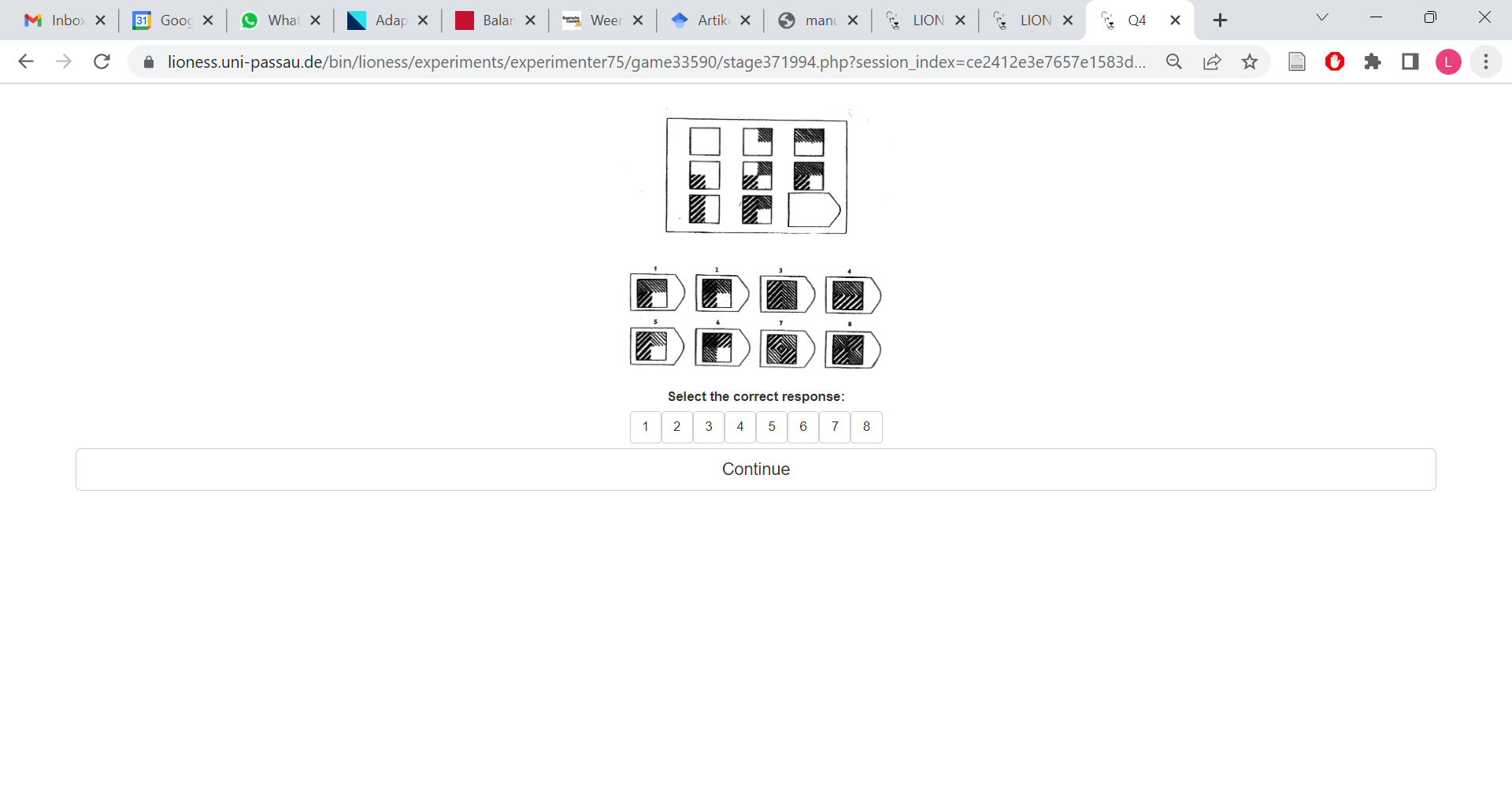


===


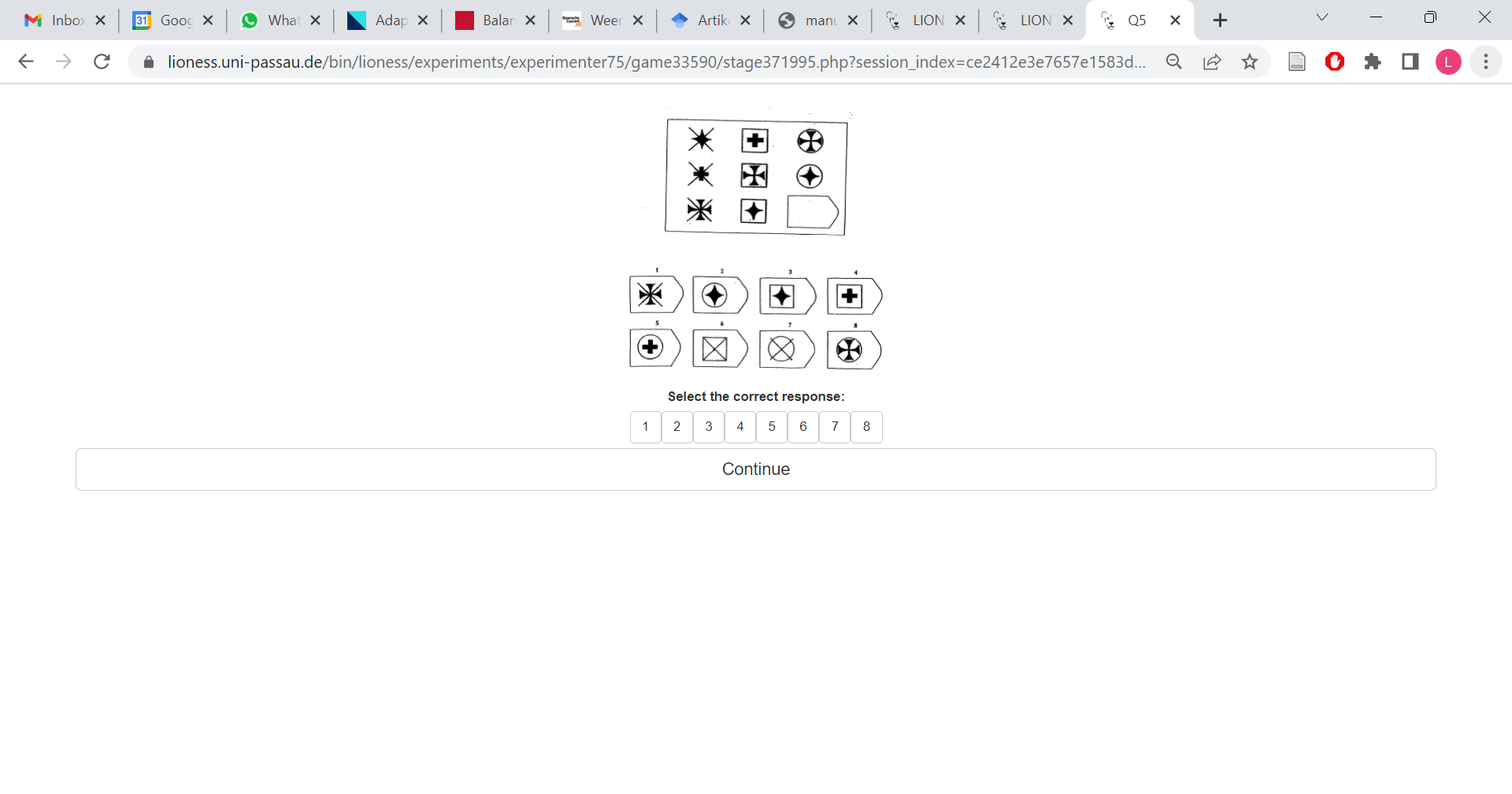


===


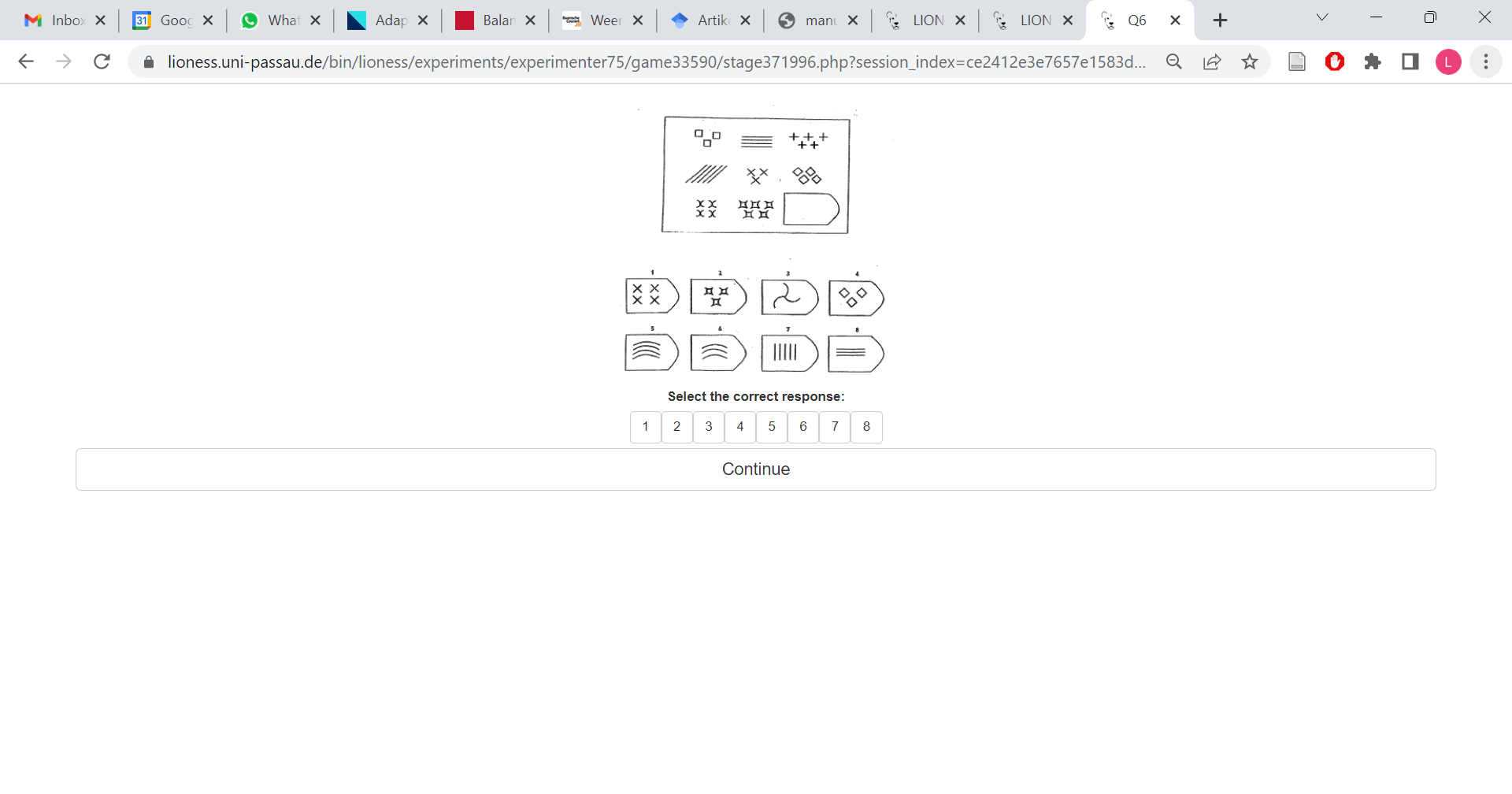


===


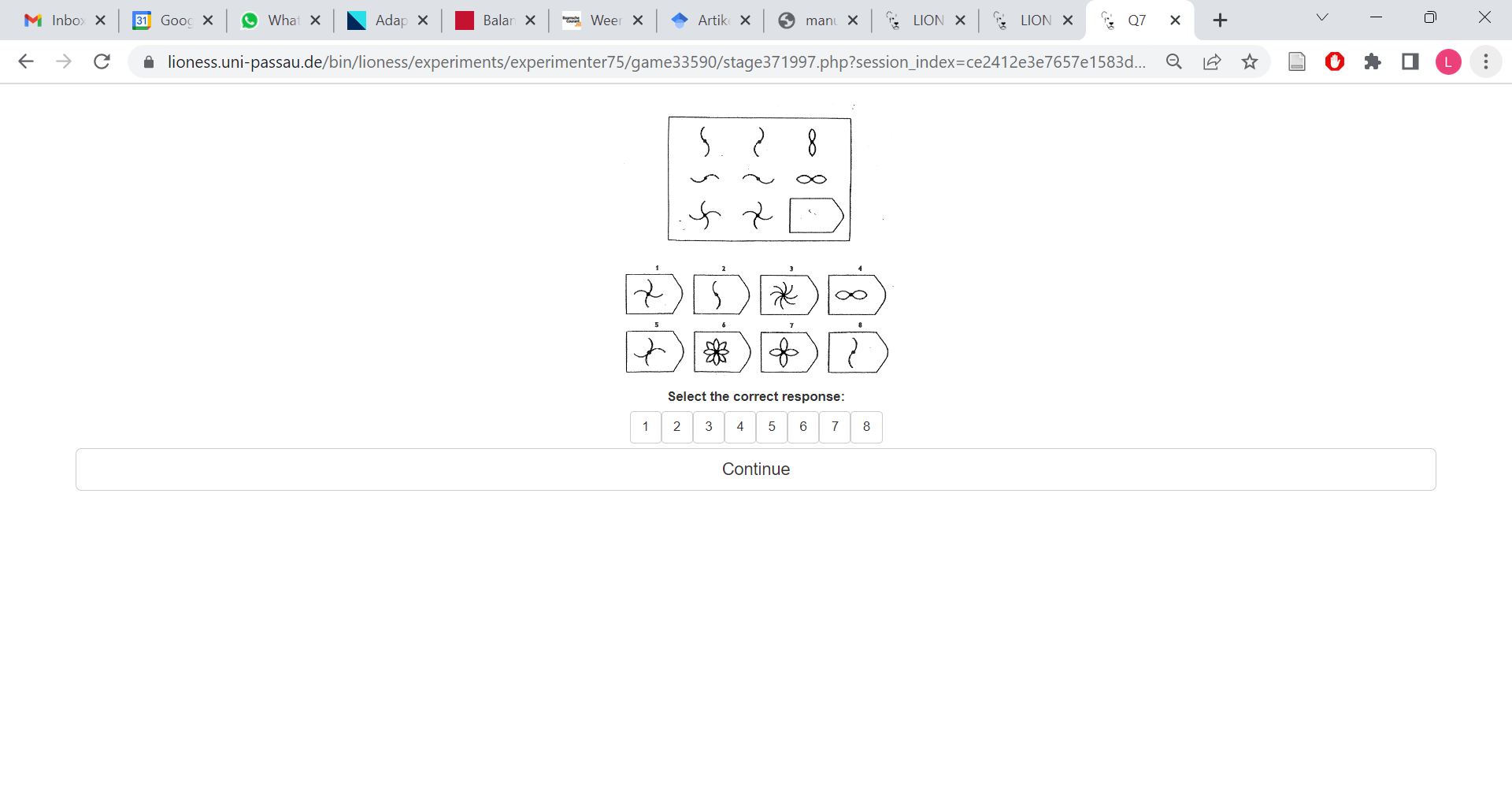


===


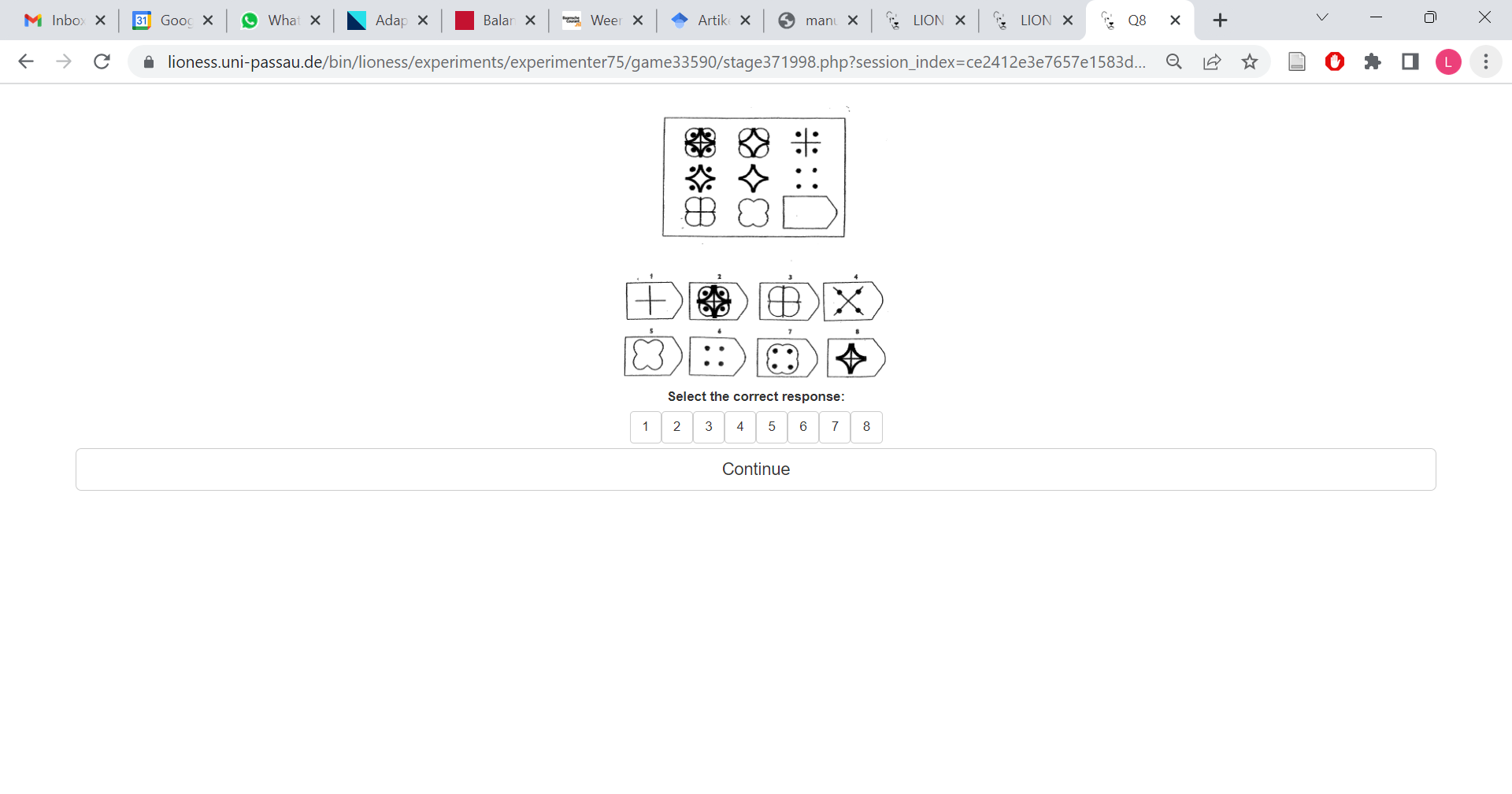


===


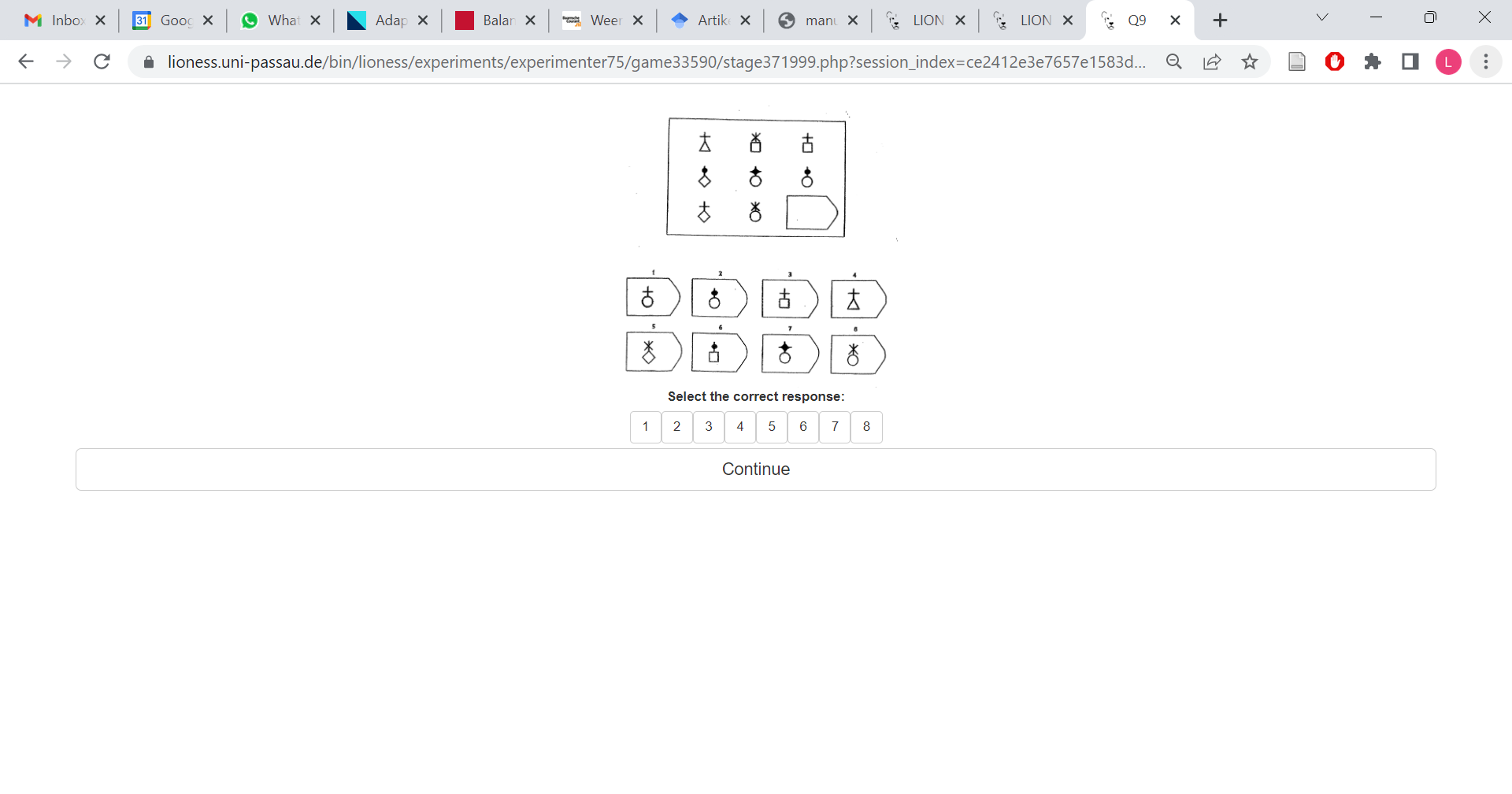


===

You have finished this test. You gave **4** correct responses, which are worth a bonus of 10 cents each. During this HIT, you have until now earned an overall bonus of **$0.40**. Please click 'continue' to get to the next test.

[Continue]

===

| After completing the questionnaire , the participants were directed to an ‘n-Back’ task. |
| --- |

# **Instructions**

In this task, you will see a sequence of thirty numbers presented one at a time.

Your job is to respond by pressing the **up arrow key when the number matches** the number that occurred **2 trials before**. If the current number does not match the number 2 trials earlier, press the down arrow key.

For example, if you saw the sequence: 3...5...2...3...4...1...4, the correct responses would be to press the up arrow key on the last "4" and the down arrow key for all other numbers.

**For each correctly identified match you will win 5 cents!** But watch out: for each false alarm you will lose 5 cents. A false alarm is when you press the arrow up key but the current number does not match the number 2 trials earlier.

Your overall bonus for this task cannot become negative.

[Continue]

===

| The n-back task showed the number sequences. |
| --- |

===

| After completing the n-back task, there was a brief exit questionnaire |
| --- |

# **Some information:**

Before you finish the experiment, we would like to ask some questions about you. This information will be anonymized and used merely for the purpose of categorizing the data.

Please select your **gender**:

[female] [male] [would rather not say]

Please insert your **age**:

[ numeric input]

[Continue]

===

| On this screen, participants had to indicate their agreement to the following statements, on a 5-point scale from ‘Completely disagree’ to ‘Completely agree’. |
| --- |

Please indicate to what extent you agree with each of the below statements.

Am the life of the party.

Sympathize with others' feelings.

Get chores done right away.

Have frequent mood swings.

Have a vivid imagination.

Don't talk a lot.

Am not interested in other people's problems.

Often forget to put things back in their proper place.

Am relaxed most of the time.

Am not interested in abstract ideas.

Talk to a lot of different people at parties.

Feel others' emotions.

Like order.

Get upset easily.

Have difficulties understanding abstract ideas.

Keep in the background.

Am not really interested in others.

Make a mess of things.

Seldom feel blue.

Do not have a good imagination.

[Continue]

===

| On this screen, participants had to indicate their agreement to the following statements, on a 5-point scale from ‘Completely disagree’ to ‘Completely agree’. |
| --- |

Please let us know about your current state and mood.

I am tired.

I am in a happy mood.

I feel hungry.

I feel stressed.

I feel awake.

I feel worried.

I am bored.

Am relaxed most of the time.

[Continue]

===

## **Final questions**

Please let us know about any problems or complaints you may have about this HIT.

For example, tell us whether you experienced technical problems, whether it was hard to understand the task, or whether the task took shorter or longer than you expected.

[open text]

===

# **Thank you!**

###

You have finished the experiment. To collect your payment for the HIT you just completed, please fill out the below code on MTurk.

**1000103**Once you have done that, you can close this window. Your bonus will be paid separately to the flat fee for this HIT. Once you have done that, you can close this window.

**===**

| Below we show the stimulus objects used in the experiment. Each of the four games had its own unique set of objects. |
| --- |


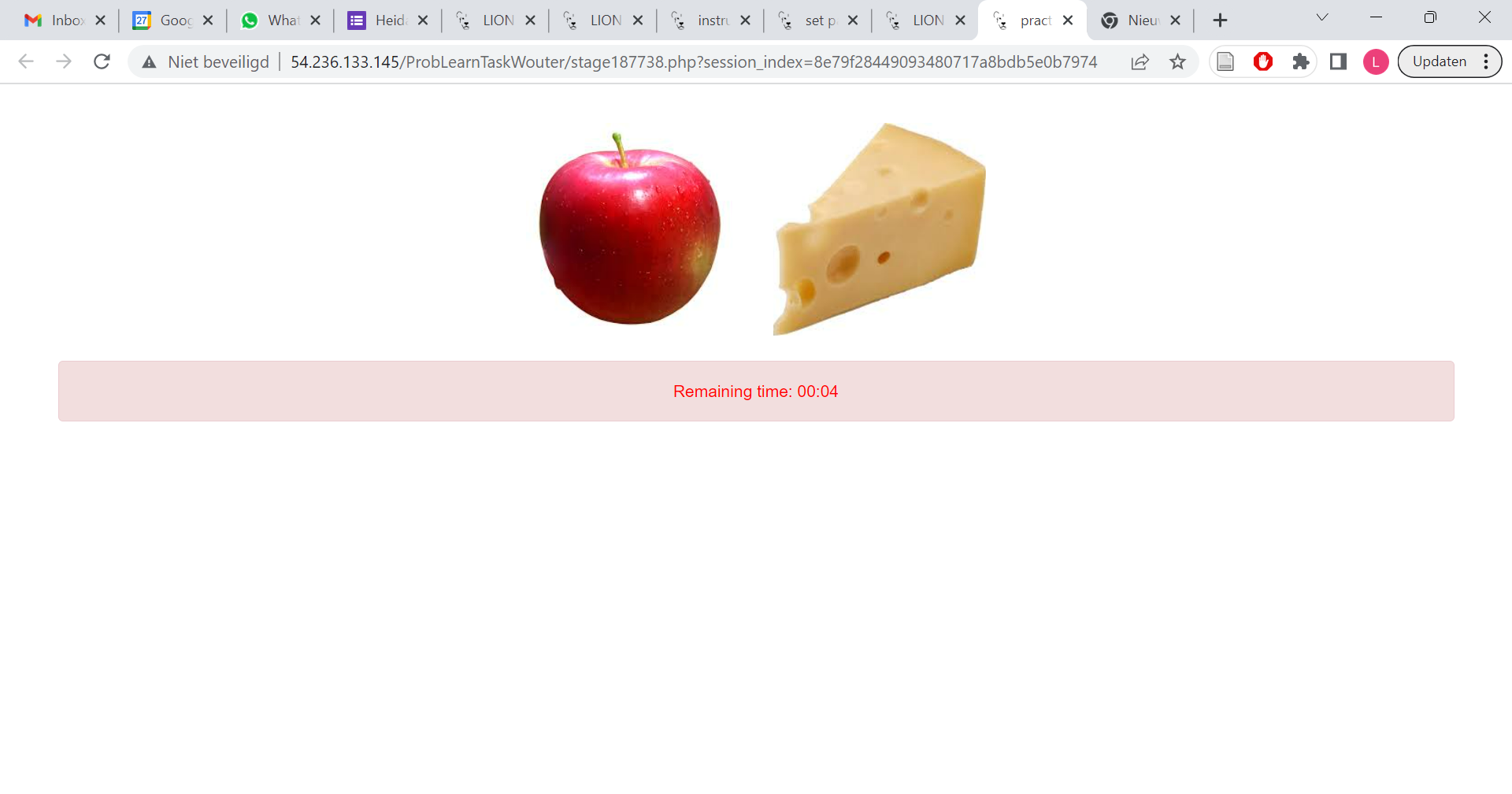

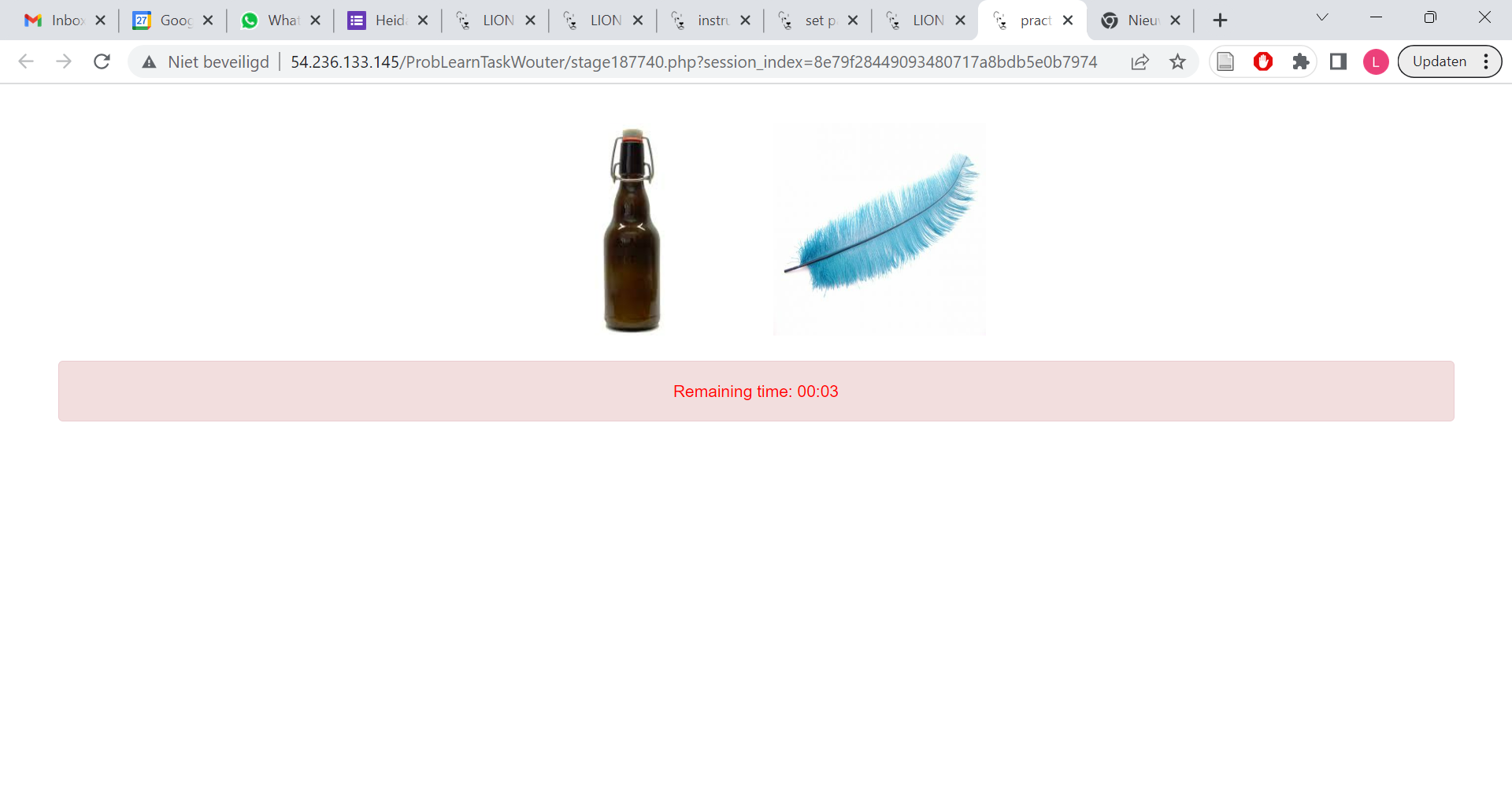

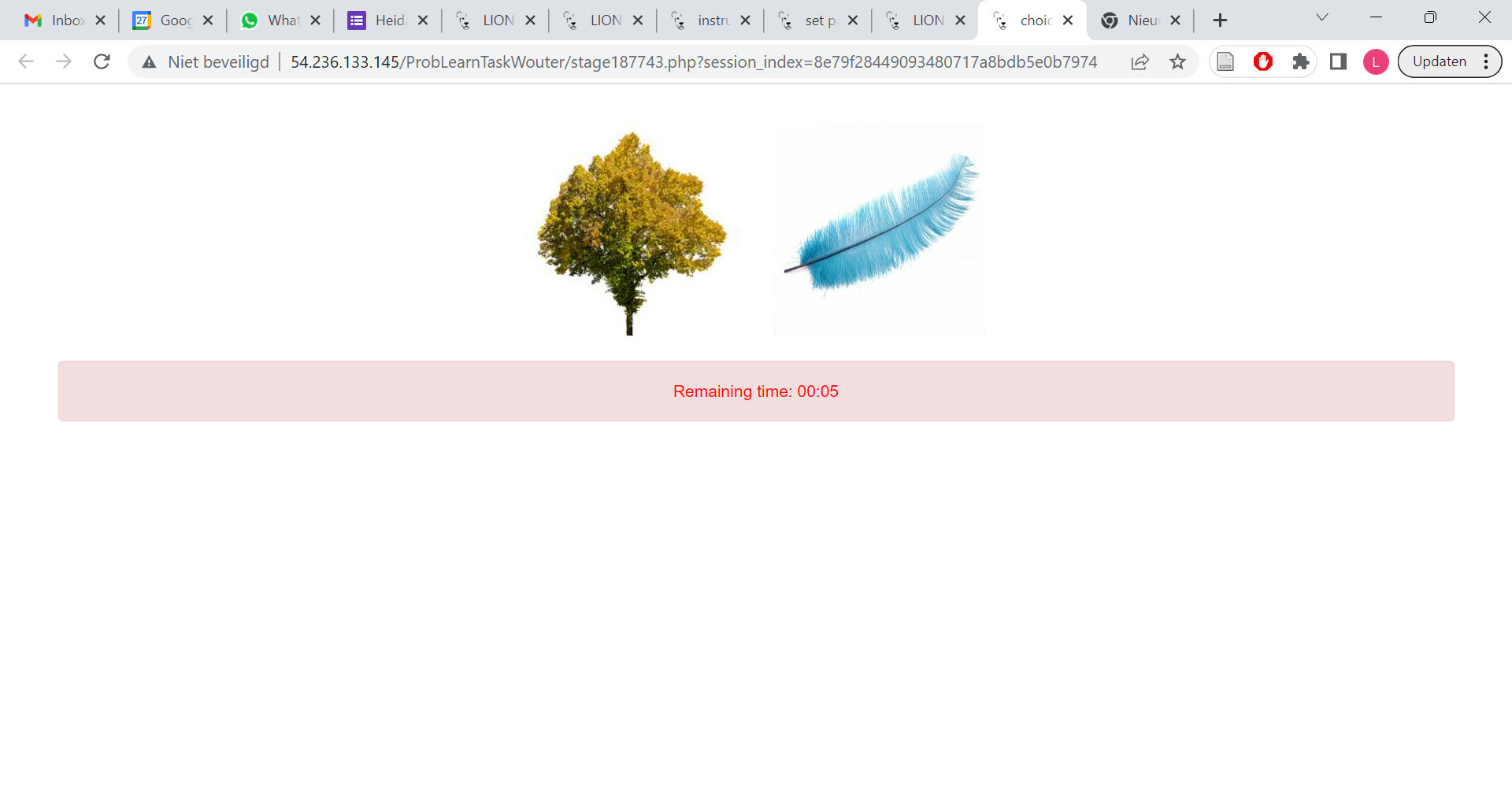


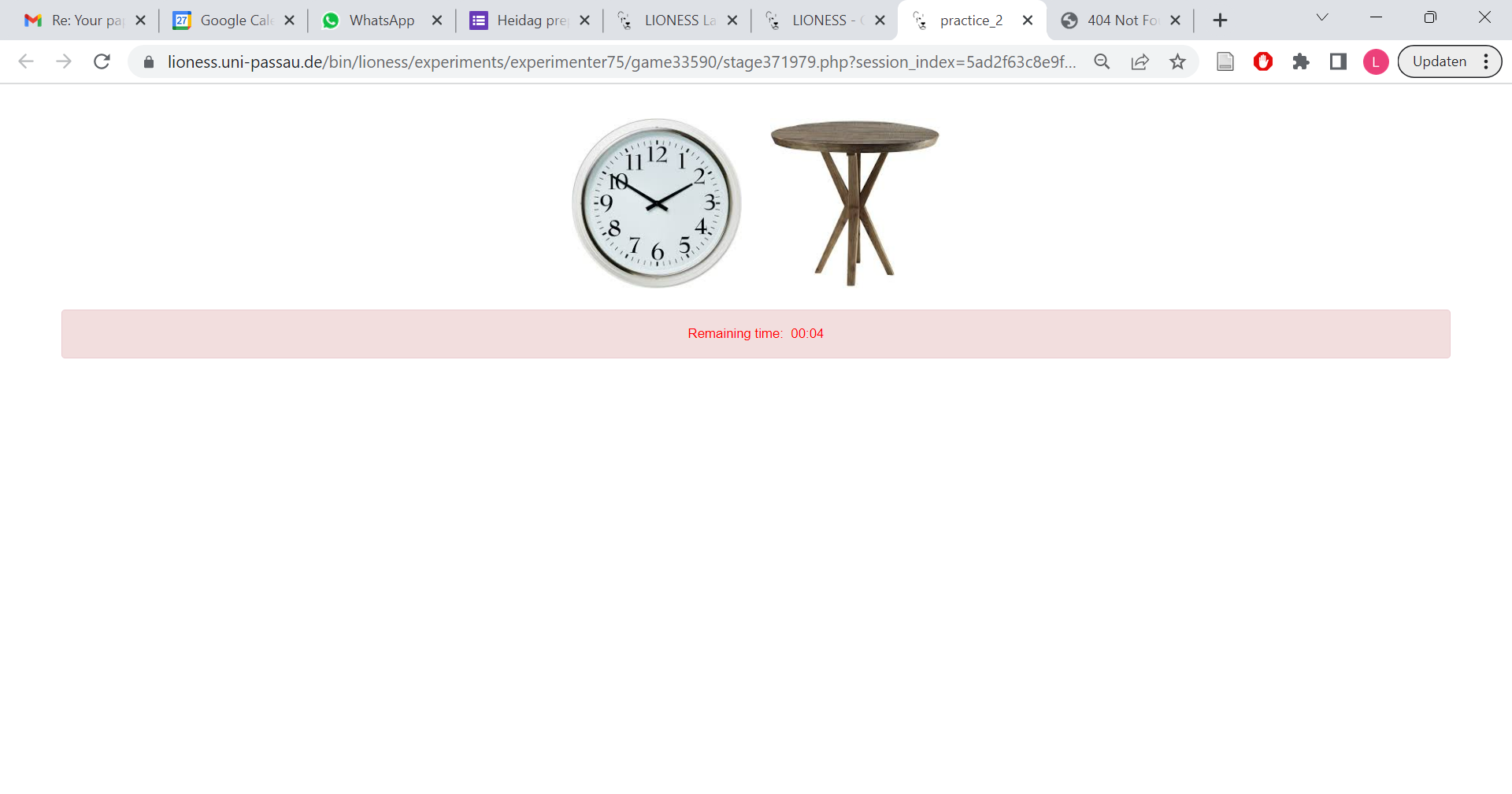

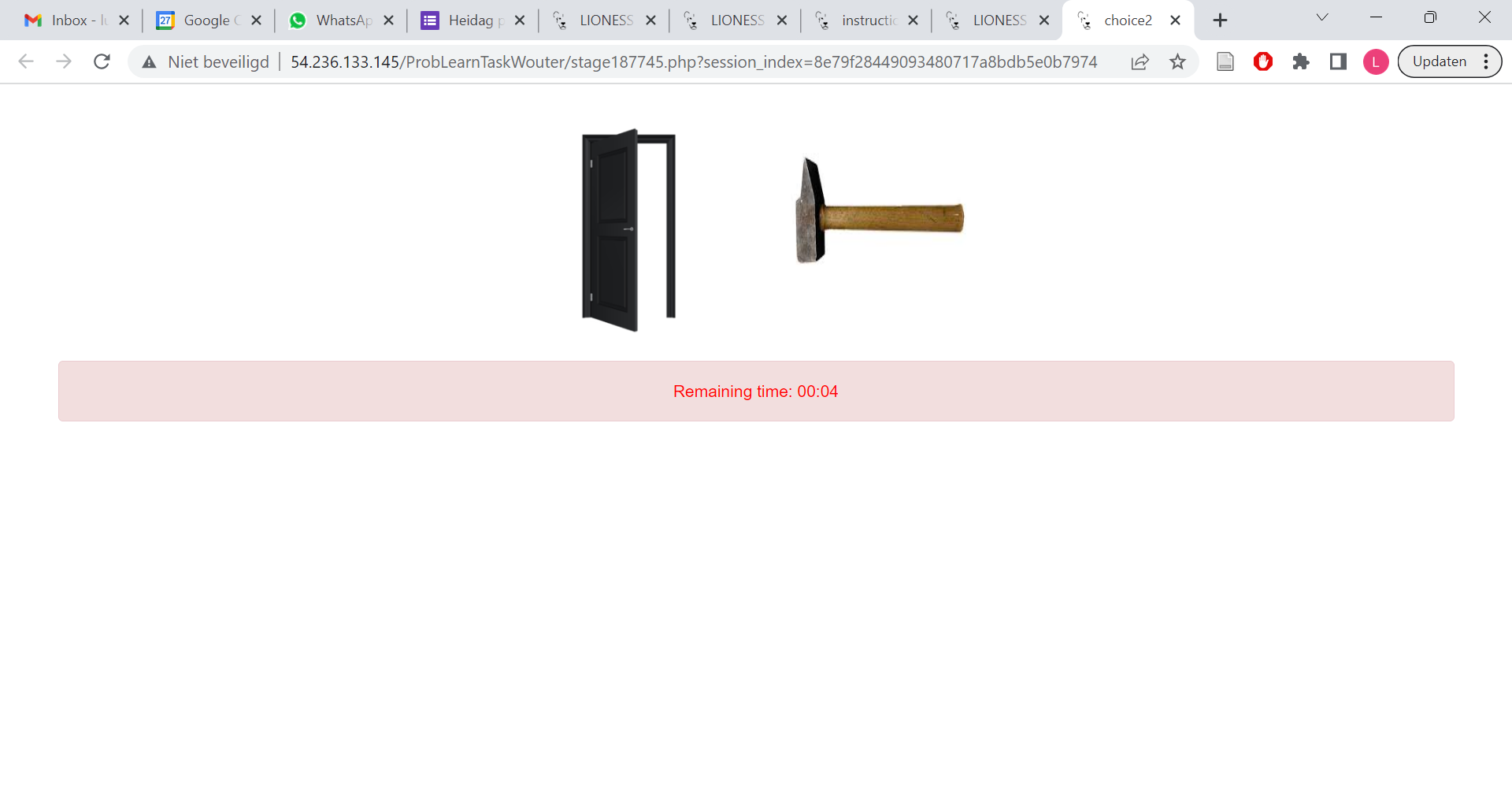

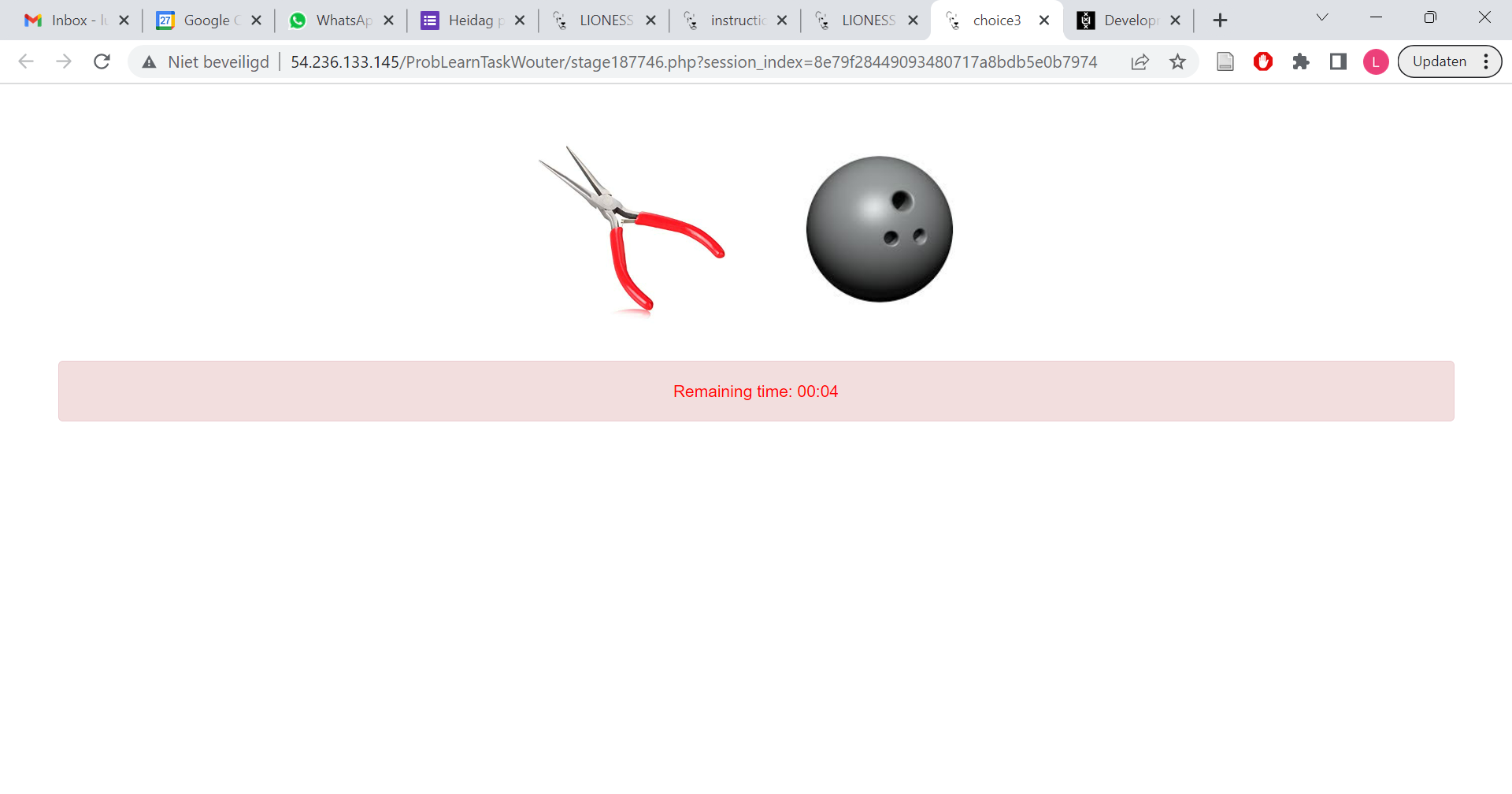


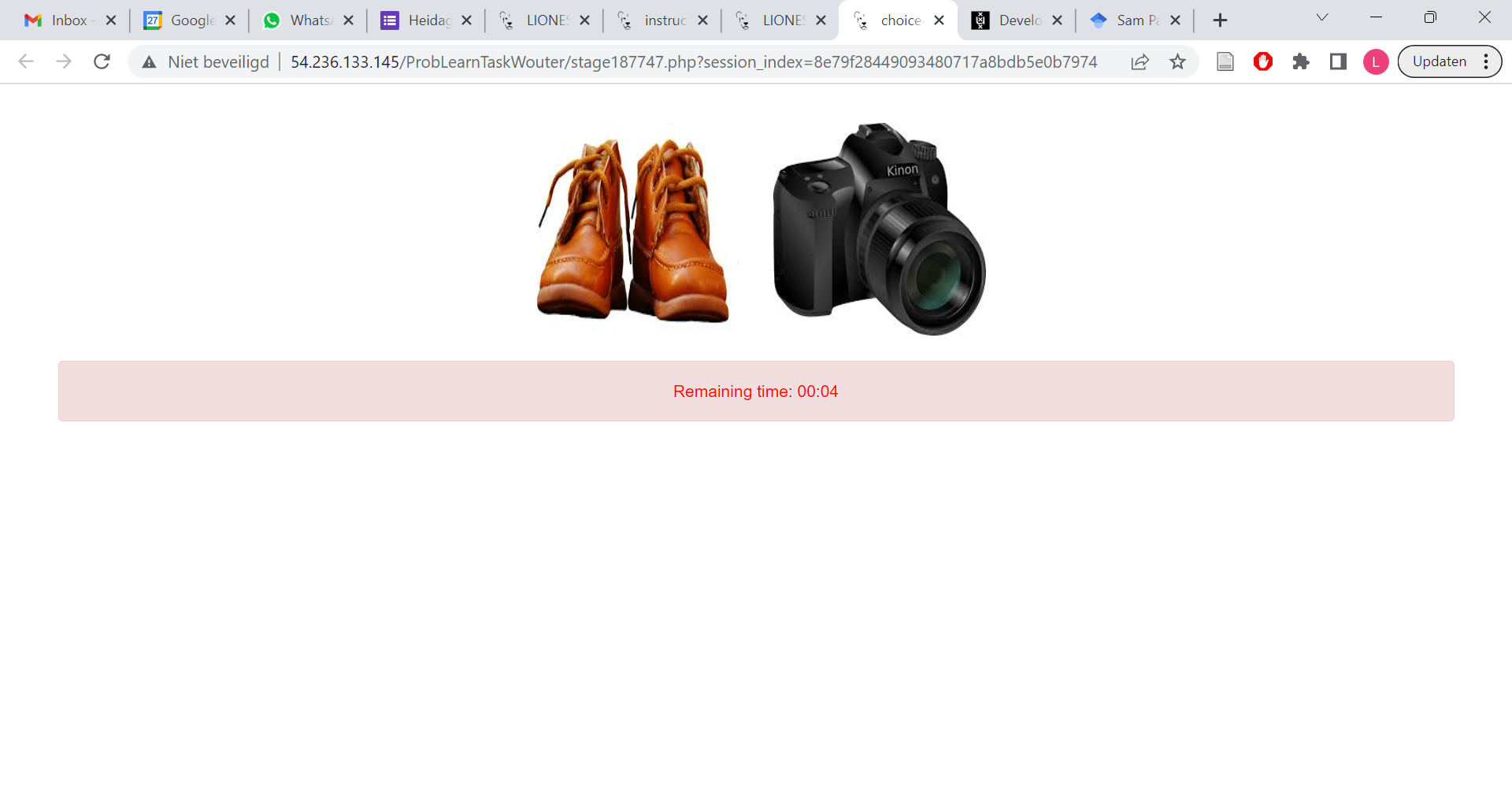

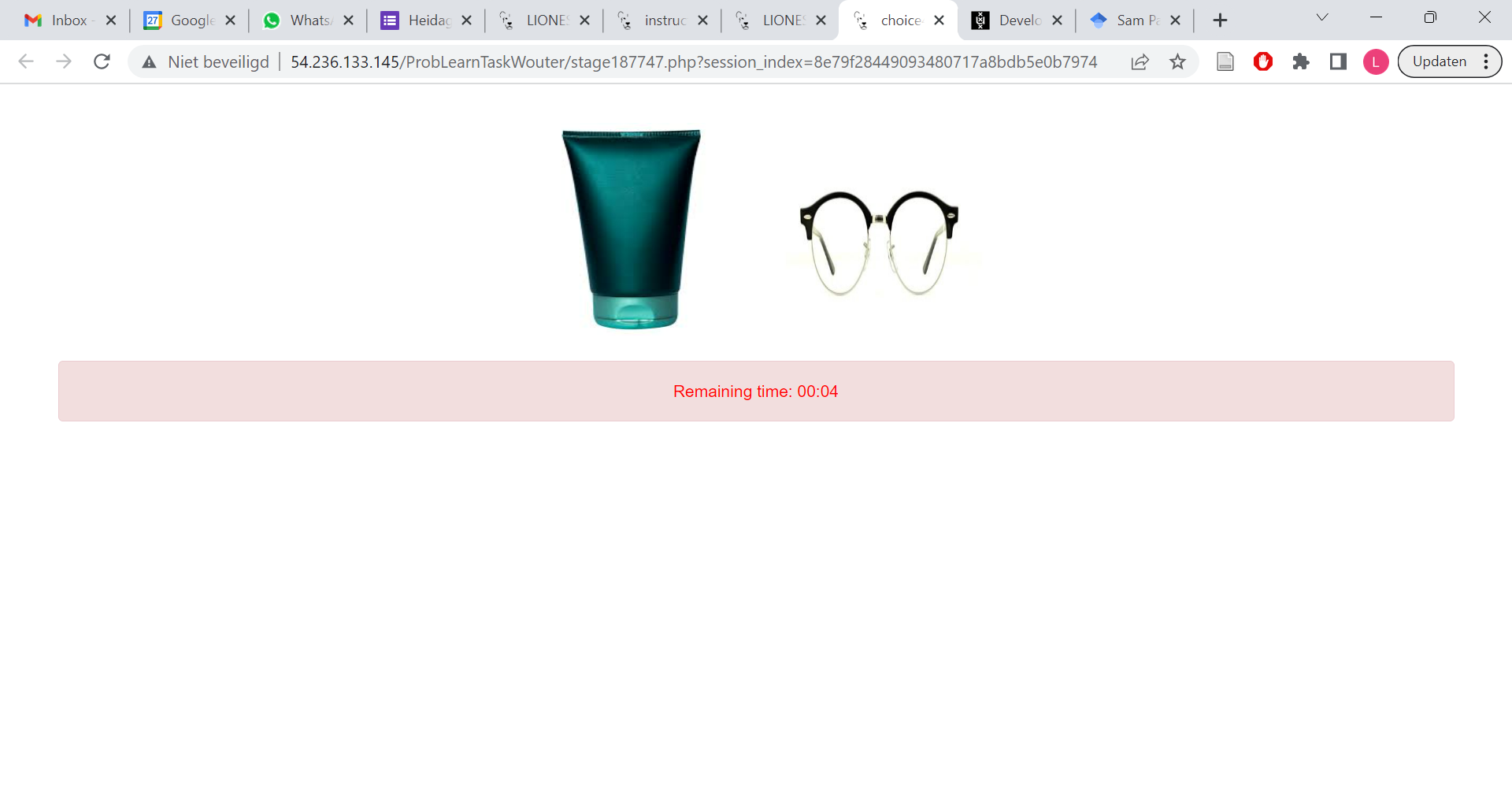

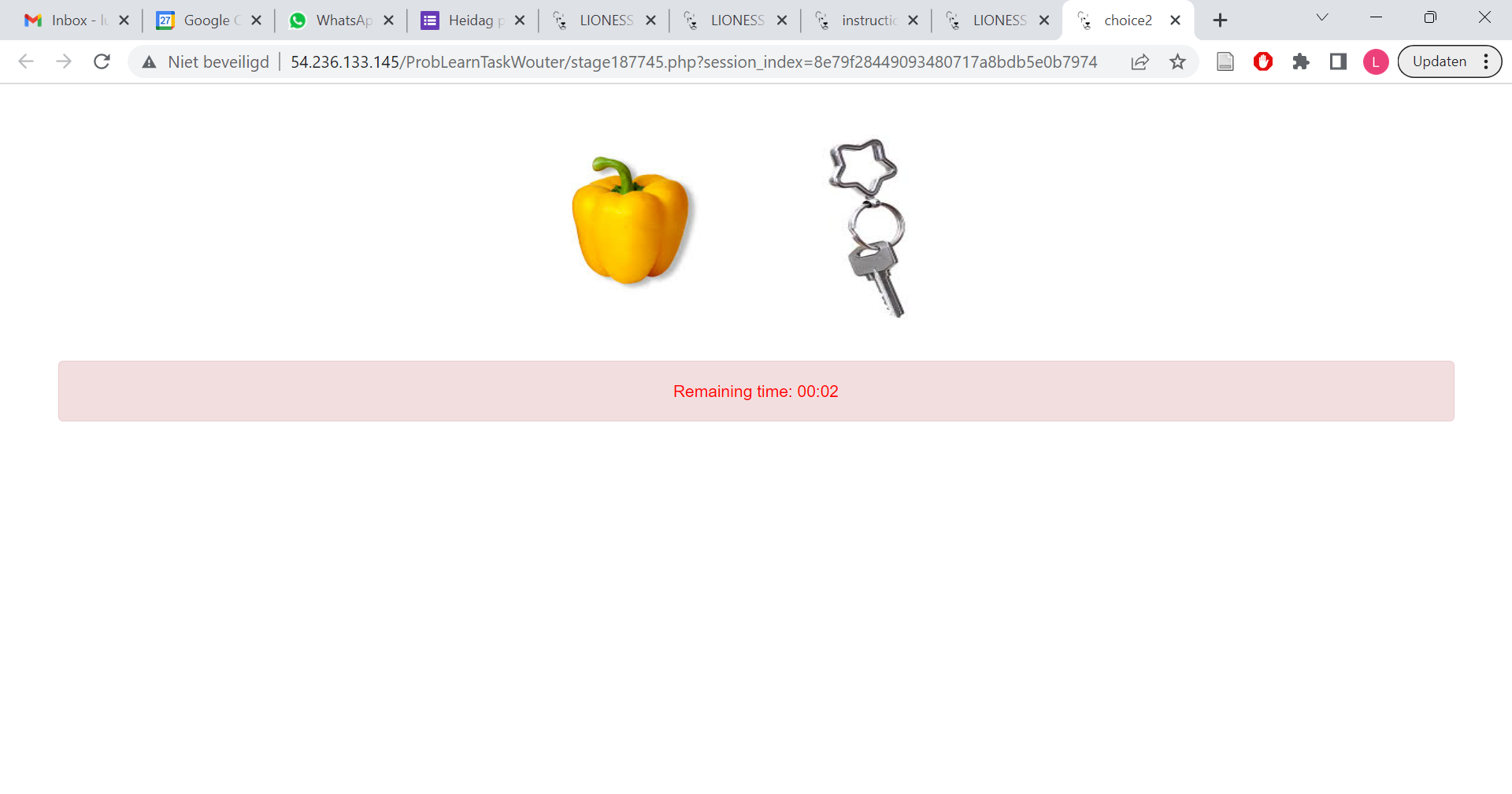

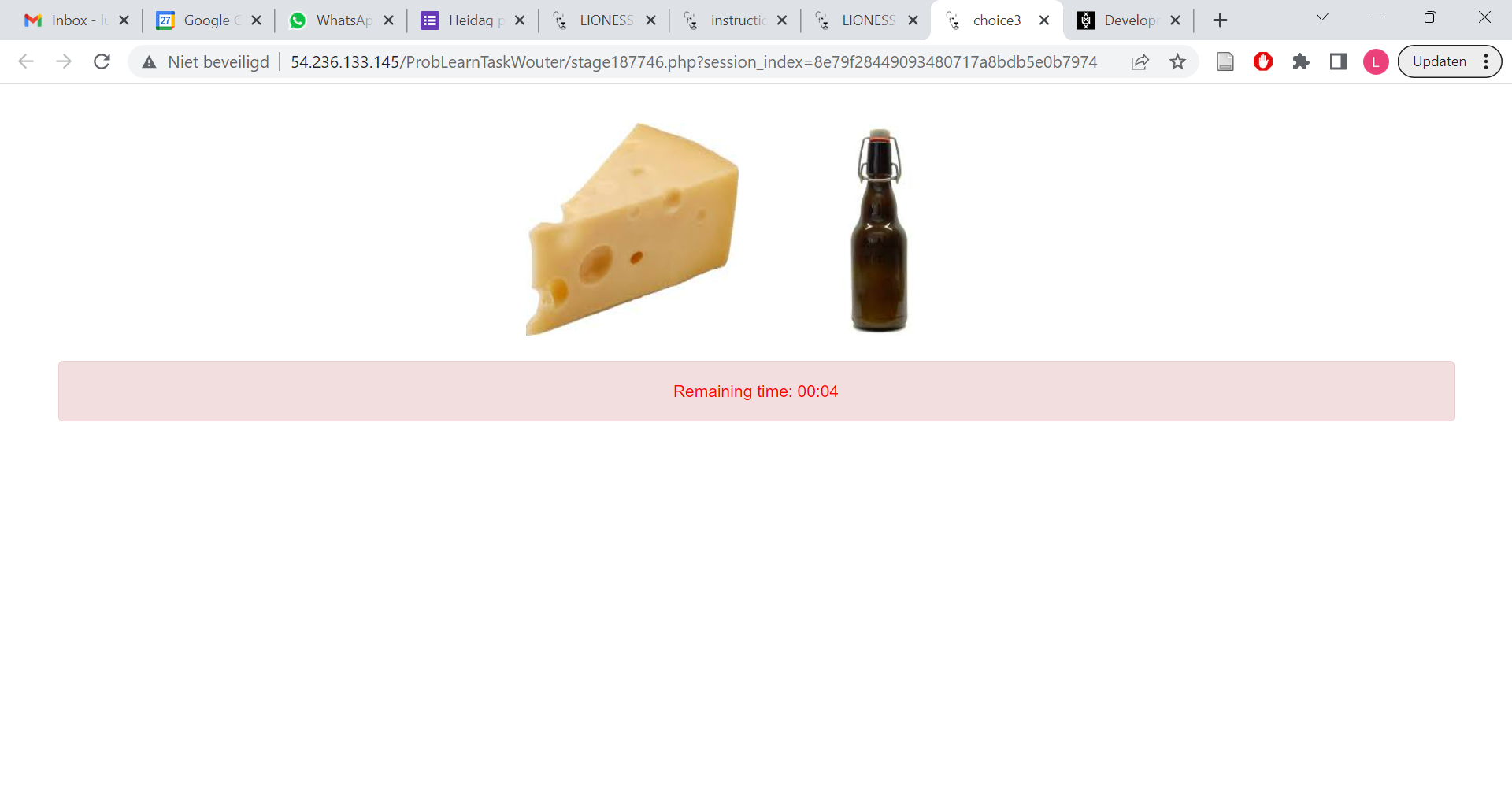

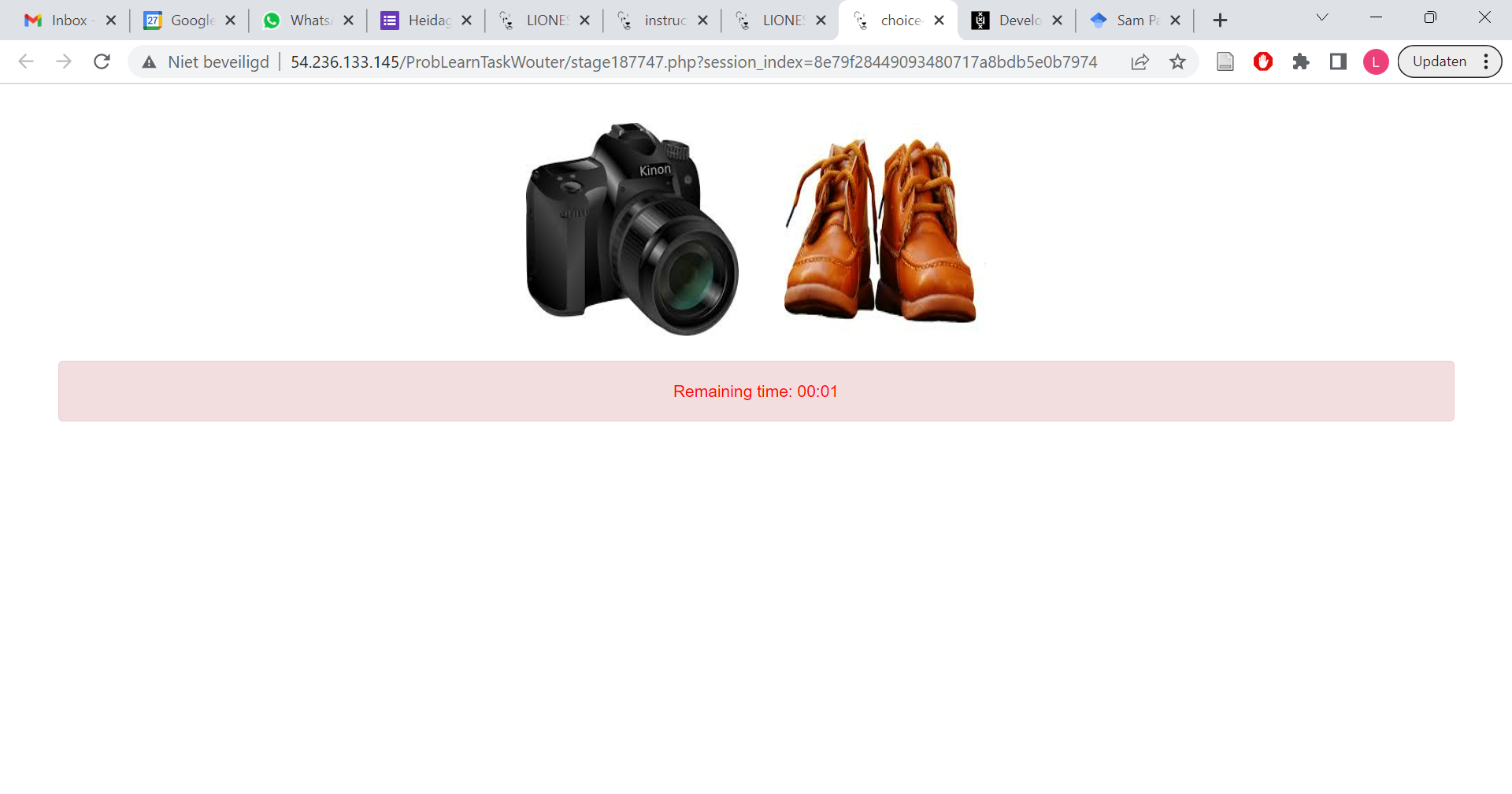


**2. REVERSAL LEARNING TASK**

# **Instructions**

Welcome! In this experiment you will have to make a series of decisions.

In each trial, you have to decide between two different pictures: a **circle** and a **triangle**, and with each choice you either win or lose. One of the symbols is **more likely to win**, and the other symbol is **more likely to lose**.

**Your task is to identify which symbol is more likely to win, and to choose that symbol.**

**
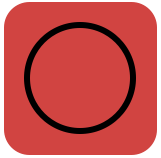

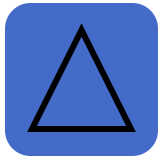
**

[Continue]

===

**Throughout the task it may change multiple times which symbol is more likely to win and which is more likely to lose.**

Keeping this in mind will help you identify the better option during each round.

**Exception rounds:**

During some rounds, it can happen that even though you choose the symbol that was more likely to win, you lose.

It can also happen that even though you choose the symbol that is more likely to lose, you still win in that round.

[Continue]

===

# **Bonus payments**

**You start with a bonus of $1.**

You can earn a bigger bonus or lose your bonus payment, depending on your choices.

**How to earn bonuses:**

After each choice you will see a sign that says whether you gained, lost, or were too late to respond in this round. Each $1 corresponds to 5 cents.

So each time you see a **"gain" picture, you have won 5 cents!**

Each time you see a **"loss" picture, you have lost 5 cents.**

If you do not respond within 5 seconds, you will see the text **"too late!" and you have lost 5 cents.**

**
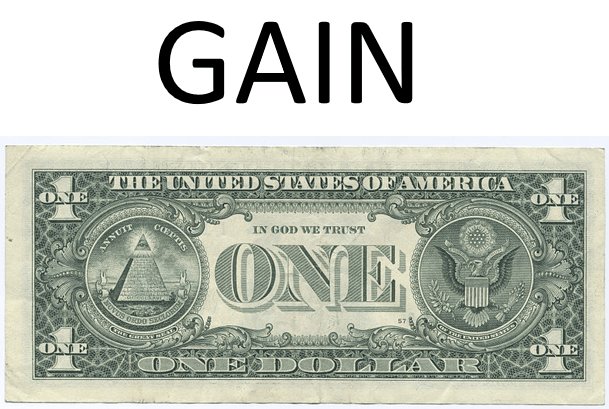
**
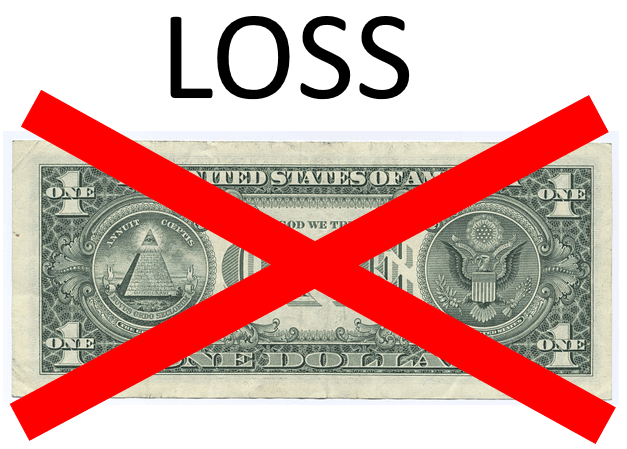

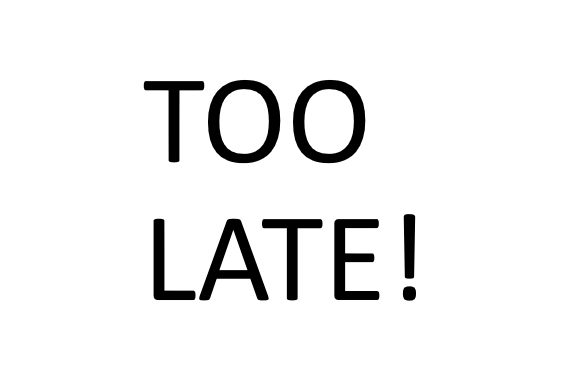


===

### **You will play 250 rounds, so the maximum bonus for this task is $12.50.**

**Your total bonus will be paid to you at the end of this HIT.** Your total bonus cannot become negative.

Remember, your **starting bonus is $1.** When you are ready to begin, click "start task".

[Start task]

===


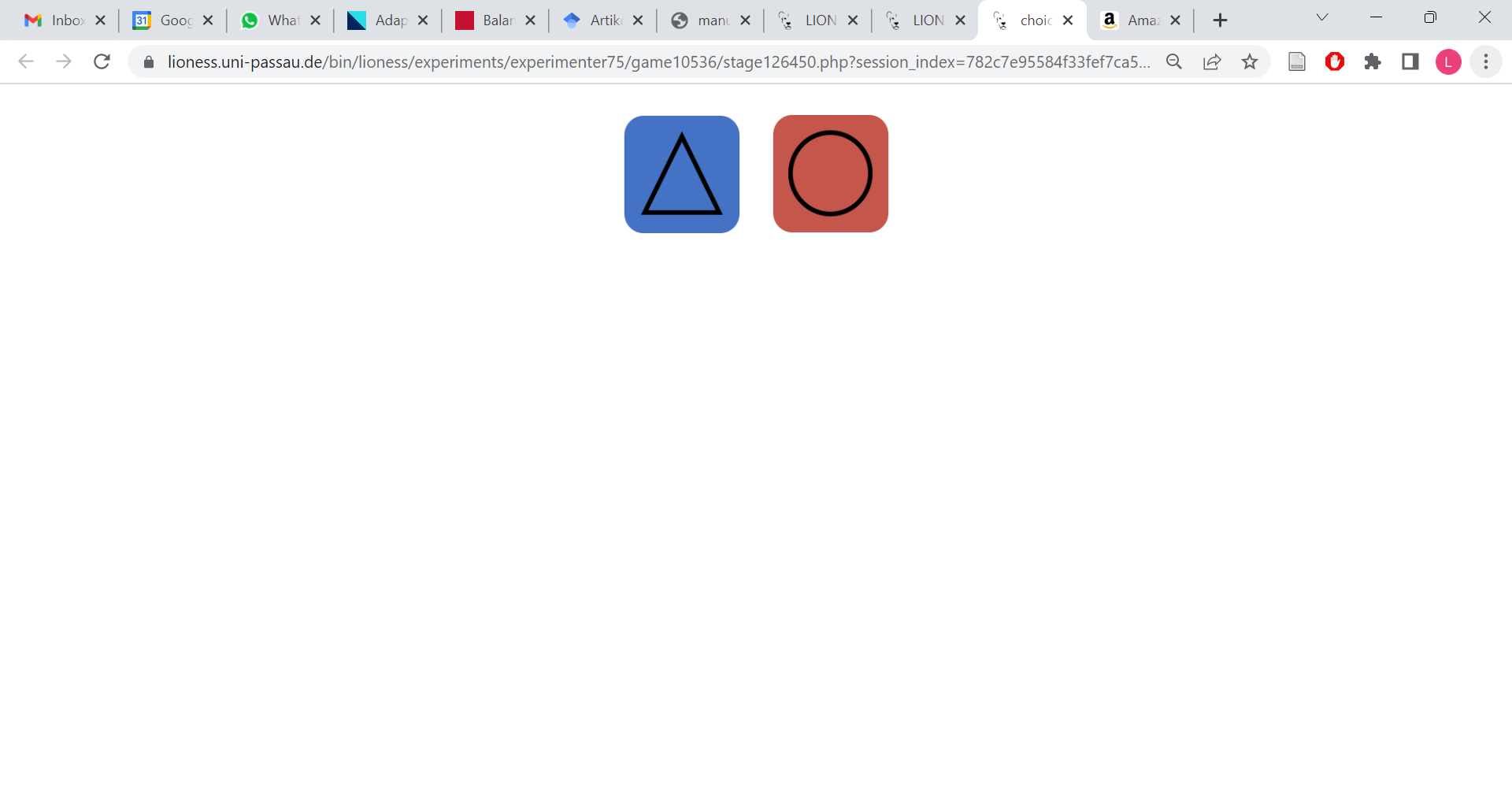


| This **decision screen** was shown for a *maximum of 5 seconds*. When the participant chose one of the options, a thick purple border appeared around the selected option, and the participant was directed to the feedback screen. If a participant had not made their choice yet after 5 seconds, they received a feedback screen reading ‘too late’ (see below). At the bottom of this description of the instructions, we show the different stimulus objects we used. |
| --- |

===

**
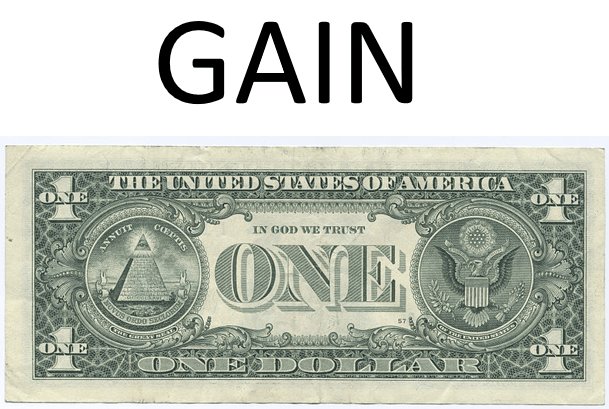
**
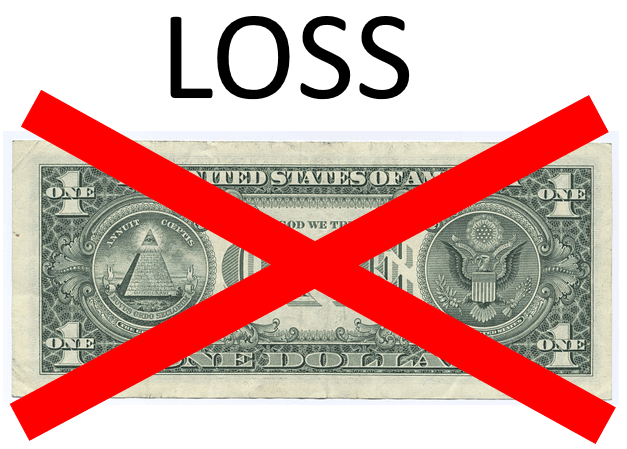

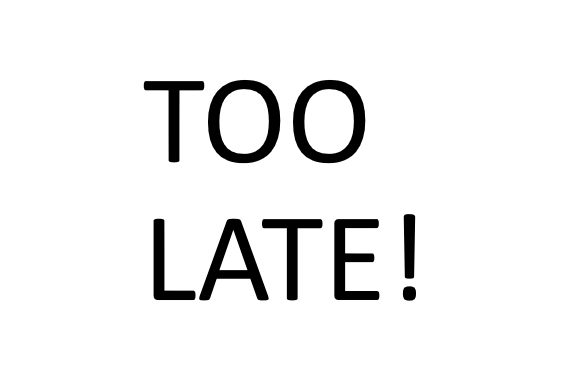


| This **feedback screen** was shown *for 2 seconds*. In each trial, it showed one of these four images, dependent on the outcome. |
| --- |

===

| After 250 trials, the task was over. |
| --- |

### **Bonus earnings**

Your earnings are calculated by adding up the outcome from all rounds you played. Your total bonus is: **$0.25**.

This is your bonus for the HIT so far.

Note that any bonus you earn will be paid on top of your guaranteed participation fee.

Next, we would like to ask you a few questions and to do two more tests before you complete this HIT.

===

| As in the reinforcement learning task, the experiment finished with the following parts: (1) Raven matrices; (2) n-back task; (3) demographics; (4) personality; (5) mood; (6) participant feedback on the experiment. See above for screenshots (identical to the reinforcement learning task). |
| --- |
